# Selective Notch Frequency Technology for EMI Noise Reduction in DC–DC Converters: A Review

**DOI:** 10.3390/s25103196

**Published:** 2025-05-19

**Authors:** Yasunori Kobori, Yifei Sun, Haruo Kobayashi

**Affiliations:** 1 Division of Informatics, Bioengineering and Bioscience, Maebashi Institute of Technology, Maebashi 376-0816, Japan; 2 College of Information Engineering, Shenyang University of Chemical Technology, Shenyang 110142, China; t172d004@gunma-u.ac.jp; 3 Division of Electronics and Informatics, Gunma University, Kiryu 376-8515, Japan

**Keywords:** switching converter, electro-magnetic interference (EMI), noise spread spectrum, clock pulse coding, frequency notch characteristics

## Abstract

This review presents our band-selective frequency technology of Electromagnetic Interference (EMI) noise spread spectrum in the DC–DC switching converter for communication devices. The DC–DC switching converter generates electromagnetic interference (EMI) noise. To comply with EMI regulations and reduce the need for bulky filters and shielding, noise spread spectrum technology is commonly employed. However, conventional methods may allow noise to encroach upon the signal band. To address this issue, selective notch frequency technology has been developed. This technology creates notch characteristic spectrum bands, ensuring a low noise level within the received frequency range. It detects the received frequency and generates a notch band there using a switching pulse control technology. This technology employs pulse coding techniques, including pulse width coding, pulse phase coding, and a combination of pulse width and phase coding. Then, we demonstrate a technique that tunes the notch band frequency to the received signal one automatically. We review their underlying principles, theoretical analyses, and experimental results, which validate the effectiveness of the selective notch frequency technology. Also, possible applications of this technology to sensor systems are discussed.

## 1. Introduction

Nowadays, switching power supply circuits are widely used in many electronic devices, thanks to their advantages of high efficiency, continuously variable output voltage, small size, and light weight [1,2,3,4,5]. Also, the communication circuit has been enhanced for improved performance and high-density packaging. However, since the switching power supply circuit is driven by a high-frequency clock, it generates some amounts of switching noises called EMI noises, and their reduction is very important [6,7,8,9,10,11,12,13,14,15,16,17,18,19,20,21,22,23,24,25,26,27,28,29,30,31,32,33,34,35,36,37,38,39,40,41,42,43,44,45].

In this review paper, first we explain the fundamental DC–DC switching converters employing the Pulse Width Modulation (PWM) control. Then, we show their EMI problems: they cause EMI noises at the clock and harmonics frequencies, and for their reduction, the clock modulation is often utilized by shaking its phase or frequency. In typical scenarios, this technology redistributes noise power across different frequencies, causing an increase in the baseline level, which sometimes causes a problem that the noise is also redistributed in the signal band, such as the AM and FM bands of the radio.

Next, we review our band-selective EMI spread spectrum technique, which realizes both a reduction in the spectrum line noise and the generation of the low noise spectrum band like the notch filter at the receive frequency band of communication devices. Furthermore, its possible applications to sensor systems are discussed.

This paper is organized as follows: In Section 2, the configuration and operation of fundamental DC–DC converters are reviewed. In Section 3, the problems of EMI in switching converters are introduced. In Section 4, conventional methods of EMI reduction with suppressing diffusion are explained, including frequency modulation with an analog spread spectrum clock generator and digital frequency modulation with a linear feedback shift register (LFSR), with the bit-inverse and bit-exchange. In Section 5, select notch band methods with pulse coding control are reviewed, including pulse width coding, pulse phase coding, pulse width, and phase coding. Then, in Section 6, the automatic notch generation method is presented, and in Section 7, its implementation and experimental results are shown. Section 8 discusses possible applications of the reviewed technology in sensor systems. Finally, Section 9 provides the conclusion.

**Remark:** the selective notch frequency characteristics demonstrated here were tested using prototype circuits built with discrete components rather than integrated circuits (ICs), and the operation and effectiveness were verified. It is important to note that this study is based on well-established switching control methods commonly used in DC–DC converters, which have already been implemented through IC integration. The additional circuitry required to achieve the research outcomes consists entirely of enhancements to the internal feedback control circuits within existing switching power supply ICs. Consequently, while IC integration leads to a slight increase in internal circuitry, it does not result in an increase in IC pin count, making it feasible to realize selective notch frequency characteristics through IC implementation. Then, electrical issues such as ESD, EMS, and input–output voltage levels are comparable to those of conventional DC–DC converters. Additionally, any increase in price or size is anticipated to be negligible.

## 2. Fundamental DC–DC Switching Converters [1,2,3,4,5,6,7,8]

There are three fundamental DC–DC switching converters: buck, boost, and buck–boost types. They are composed of power and control stages, and their configurations are almost the same among the three types. The power stage has a switch, an inductor, a diode, and an output capacitor. However, its construction differs among them; the differences are distinguished by their positions. The control stage of each converter is quite similar, consisting of a comparator, a D-type Flip-Flop (DFF), and a reference voltage. Their voltage conversion ratio (Vo/Vi) and polarity of Vo vary, where Vi represents the input voltage, and Vo represents the output voltage: 0 < Vo < Vi for the buck converter, 0 < Vi < Vo for the boost converter, and 0 < −Vo ≷ Vi for the buck–boost converter.

### 2.1. Buck Converter with PWM Control

**Figure 1:**
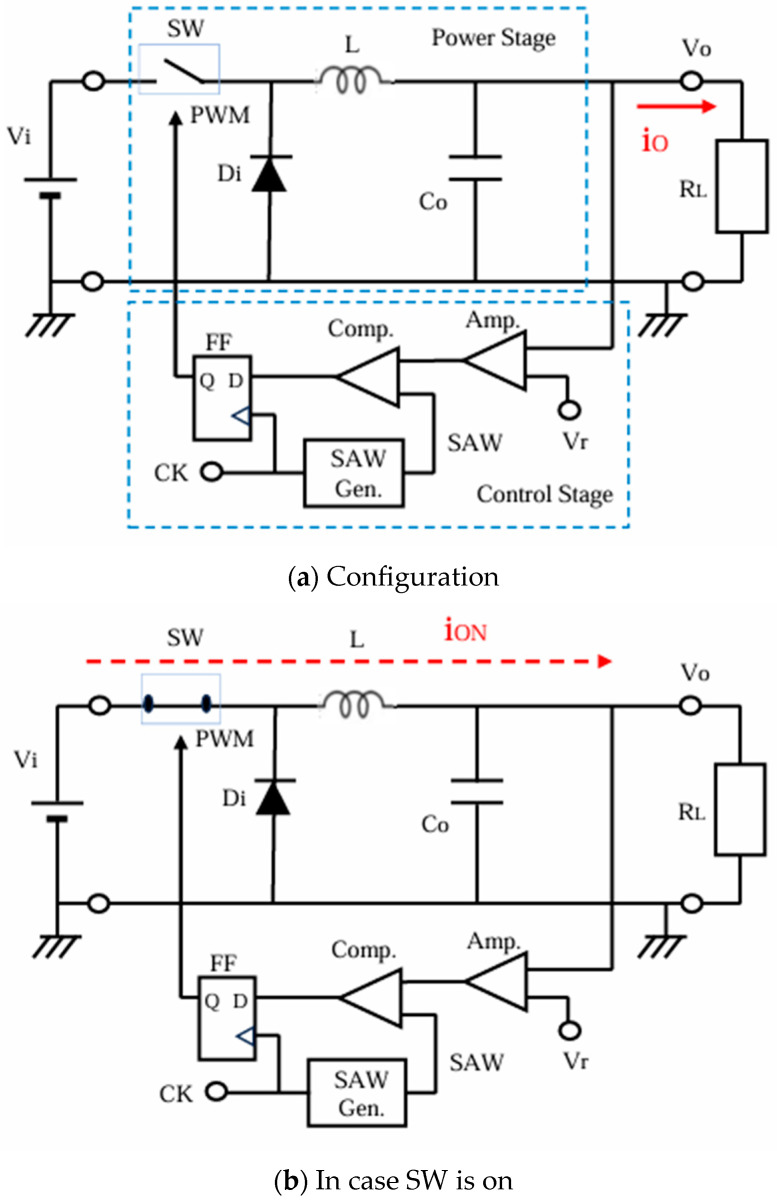

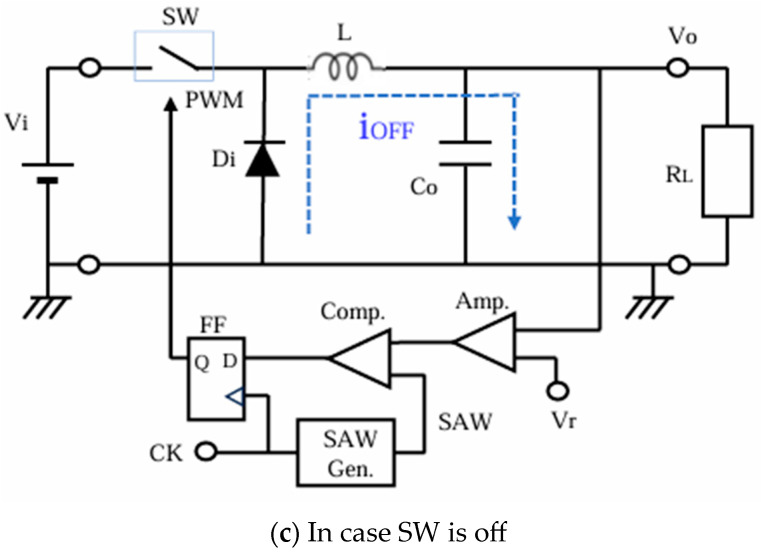
Configuration and operation of the buck converter.

Figure 1 shows the configuration and operation of the buck converter. The power stage is composed of a switch (SW), an inductor (L), a diode (Di), and an output capacitor (Co). The control stage is composed of an amplifier (AMP), a comparator (CMP), a D-type Flip-Flop (DFF), and a sawtooth generator (Figure 1a). The red/blue broken line represents the current flow when the PWM pulse is high/low, respectively (Figure 1b,c).

**Figure 2:**
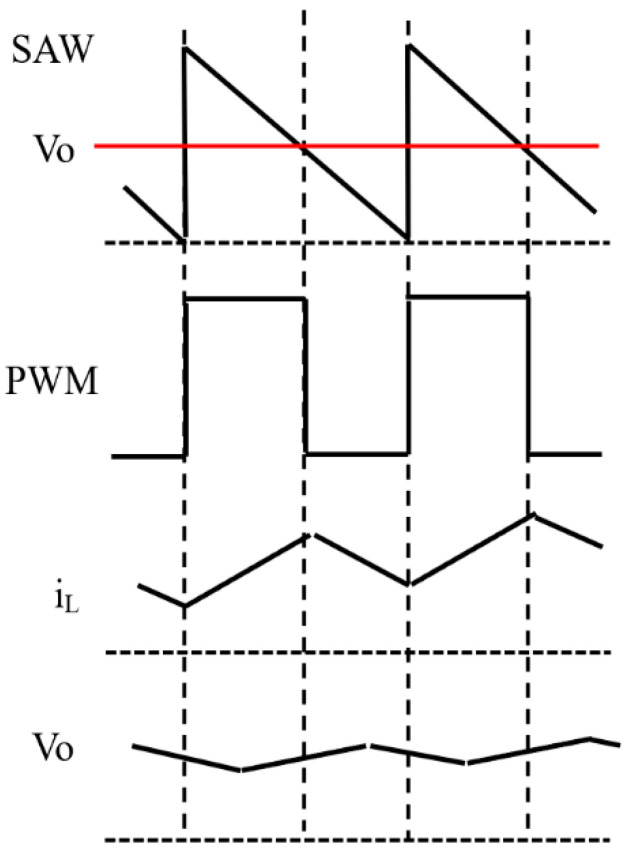
Waveforms of the buck converter.

In the power stage, SW is regulated by the PWM signal. In case the PWM signal is high, SW turns ON, and the input current from the power supply flows through SW to L (as shown by the red broken line in Figure 1b). During this period, the input current increases, flowing into Co and R_L_, resulting in an increase in Vo (Figure 2) and raising the magnetic energy in L. Conversely, when the PWM level is low, SW turns OFF, and the energy stored in L causes the current to flow through Di (as indicated by the blue dotted line in Figure 1c). Then, Vo decreases and the duty ratio (D) of the PWM signal increases.

In the control stage, the AMP amplifies the voltage error of Vo and the reference voltage (Vref). This amplified error voltage is then compared with the sawtooth signal (SAW), and the PWM signal is generated. The DFF receives these data synchronously with the internal clock (CK) to provide the SAW pulse so that the SAW signal is generated by the sawtooth generator. Vo varies based on D, and Vo is related to D as shown by the following equation:

Vo = D · Vi(1)

Vo is less than Vi due to 0 < D < 1.

### 2.2. Boost Converter

**Figure 3:**
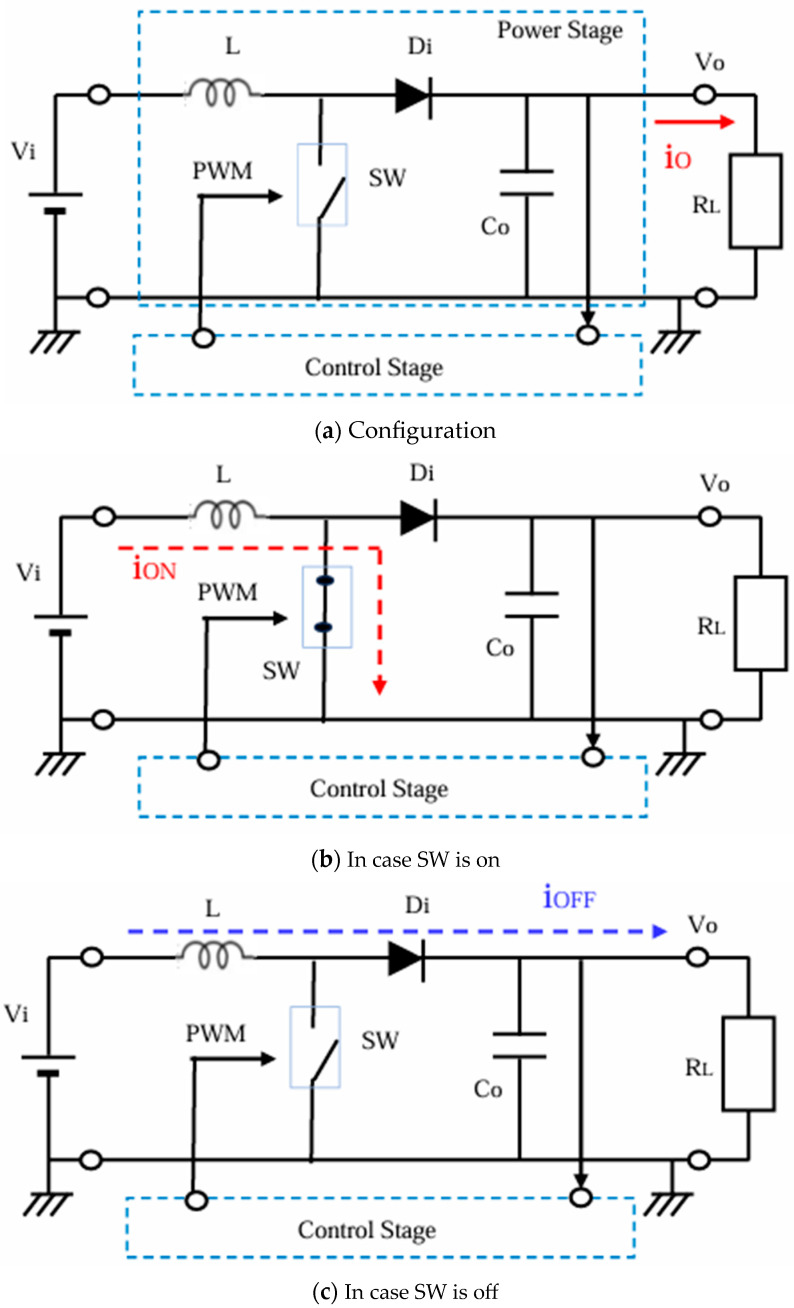
Configuration and operation of the boost converter.

Figure 3a shows the configuration of the boost converter, where the control stage is the same as the one in Figure 1. When SW is ON, the input current flows through L, and it does not flow to Co or R_L_, and then L charges the magnetic energy (Figure 3b). When SW is OFF, the current flows from L to Co and R_L_ through Di (Figure 3c). As a result, the current into Co is intermittently interrupted, leading to some output voltage ripple. Vo is expressed by

Vo = 1/(1 − D)·Vi(2)

Notice 1 < 1/(1 − D), and Vo > Vi.

### 2.3. Buck–Boost Converter

**Figure 4:**
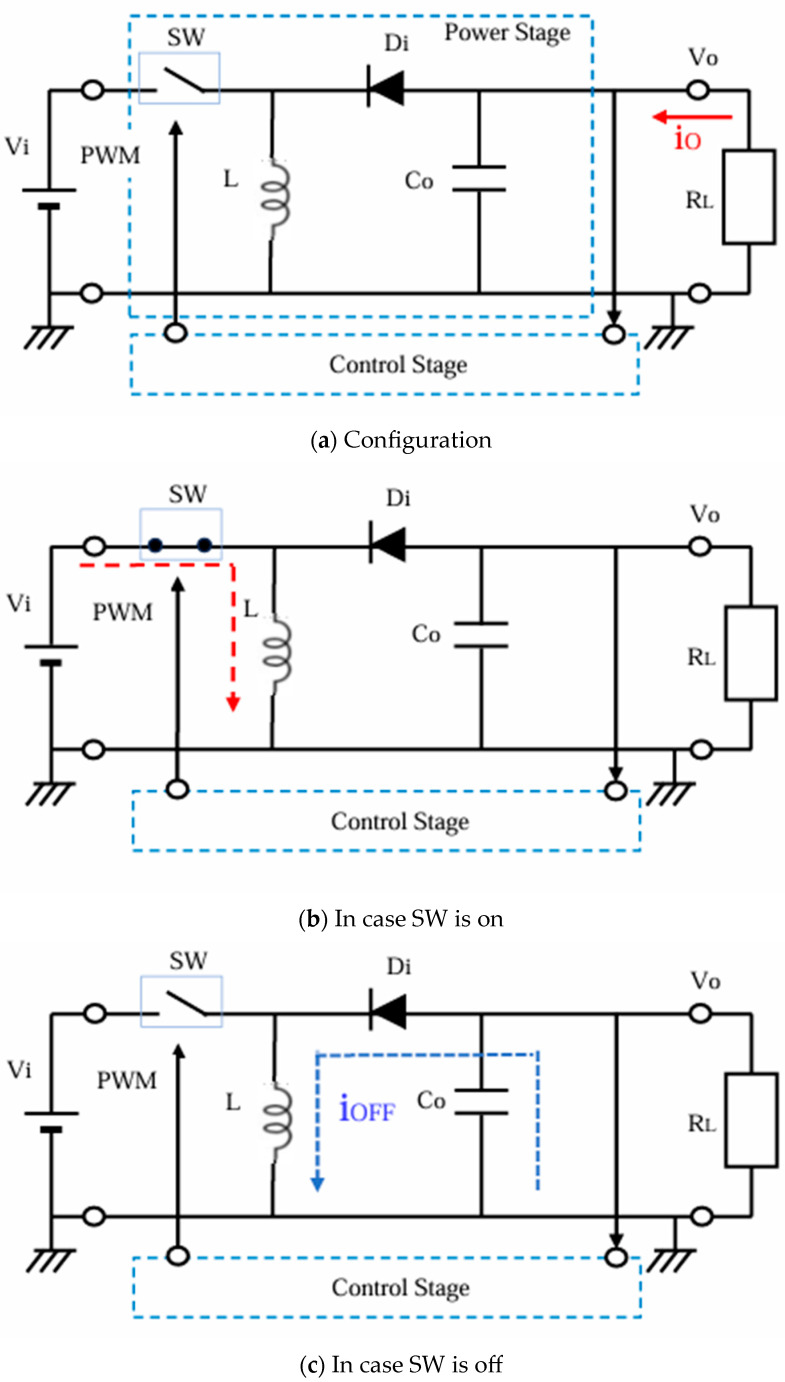
Configuration and operation of the buck–boost converter.

The buck–boost converter produces a negative voltage output. Figure 4a illustrates its configuration. It operates as follows: When SW is ON, the input current flows through SW and L (Figure 4b). When SW is OFF, the inductor current flows into Co in the opposite direction, as indicated by the blue dotted line in Figure 4c. As a result, the polarity of Vo becomes negative. Vo can be expressed by Equation (3), and the absolute value of Vo varies widely over or less than Vi.

Vo = −D/(1 − D)·Vi(3)

**Remark:**

(i) There is still room for improvement in current DC–DC converter topologies, depending on their specific applications. For example, see [34];
(ii) Another type of switching-mode converter is the switched-capacitor converter, which comprises only switches and capacitors, without the need for inductors or transformers [33]. Its features are light weight, small size, high power density, and low EMI emissions. However, it has certain limitations, such as its capacity to handle only limited output current, its output voltage being step-wise rather than continuous, and its relatively low efficiency. This paper does not discuss this type of converter because of its low EMI emissions.

## 3. EMI Problems in DC–DC Converters

In the above-mentioned three basic types of switching DC–DC converters, input voltage is stepped down or stepped up by rapidly switching the voltage and current applied to the main inductor. In this process, high-frequency currents are switched through the input power source (Vi) and its supply lines, and high-speed voltage and current switching occurs across the main switch, inductor, diode, and their wiring paths. As a result, large high-frequency currents flow through the input voltage lines and switching lines, emitting unwanted EMI noise as if they were antennas.

**Figure 5:**
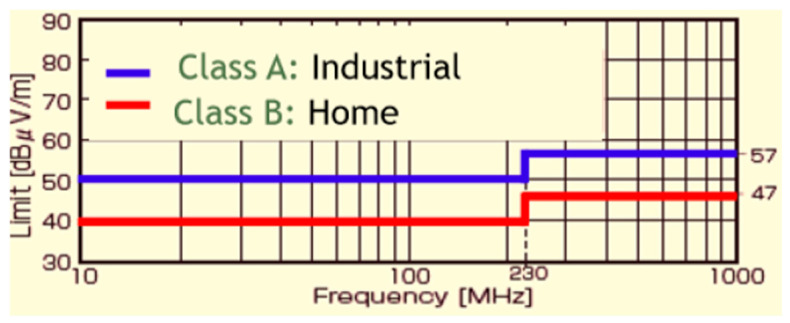
EMI regulation in Japan (CISPR22).

There are several EMI test standards, including the IEC 61,000 series, CISPR 16, EN standards, FCC standards, and VCCI. Electronic appliances must meet these standards, depending on regions and applications [43,44,45]. Figure 5 shows one example of the CISPR regulations.

**Figure 6:**
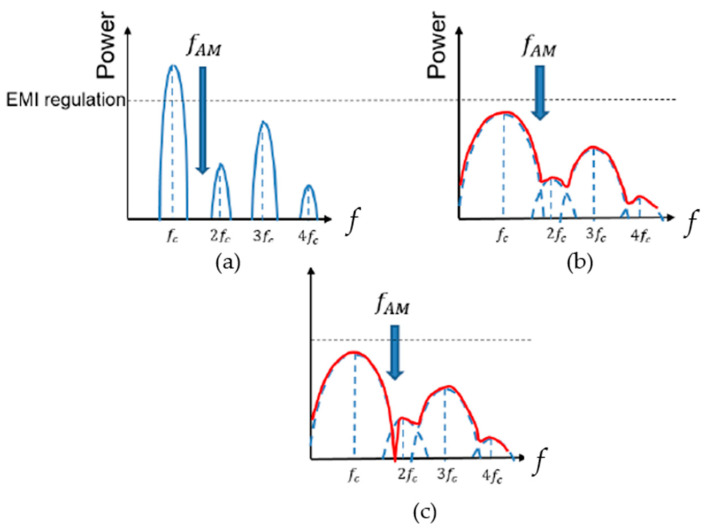
EMI from DC–DC converter. (**a**) Original spectrum without clock modulation. (**b**) With conventional noise spread spectrum method. (**c**) With band selective noise spread spectrum method.

Noise spread spectrum techniques in DC–DC converters can help meet these standards and alleviate the burden of bulky filters and shields. They are effective in reducing EMI noise caused by conduction interference, radiation interference, common-mode noise, and differential-mode noise. The EMI noise exhibits a significant line spectrum and includes substantial noise spectra extending into high-frequency bands (Figure 6a).

To address this, conventional noise-spreading techniques modulate the frequency of the clock pulse driving the power switch (Figure 6b). By dispersing the prominent line spectrum periodically, the peak levels of the spectrum are reduced. While this method effectively lowers the peak levels of specific spectra, it also spreads noise across the entire frequency range. Although this approach is effective for meeting EMI test standards, it can be detrimental to devices designed to receive weak radio signals. The noise emitted from power circuits primarily consists of harmonics and noise synchronized with the internal clock. The noise with the highest energy level is generated by the switching operation of the power switch MOS and its driving pulses. Electromagnetic noise caused by these switching operations can manifest as radiated interference noise emitted into the air from wiring or cables, or as conducted interference noise that disrupts other circuits within the system or external devices connected via the power supply line. Essentially, these interference noises can interact with other devices as differential mode noise.

The root cause of these interference noises lies entirely in the switching frequency of the clock pulses that control and drive the power semiconductors, as well as their harmonics. Therefore, we monitored and checked the EMI interference noise generated by these clock pulses. Additionally, we explored new coding techniques for these clock pulses and investigated a clock pulse control method for DC–DC switching power supplies that could produce notch characteristics with significantly reduced noise within specific frequency bands of the resulting EMI noise spectrum (Figure 6c).

## 4. Conventional Methods of EMI Reduction with Suppressing Diffusion [9,10,11,12,13,14,15,16,17,18,19,20,21,22,23,24,25,26,27,28,29,30,31,32,33,34,35]

This section reviews some spread spectrum technologies for EMI reduction with diffusion suppression of the power supply and switching noises. This technology has a long history [9,10,11,12,13,14,15,16,17,18,19,20,21,22,23,24,25,26,27,28,29,30,31,32,33,34,35]. Basic spread spectrum technologies for clock noise involve modification of the clock with frequency, phase, and pulse width. The following will illustrate the one with the coding of the clock using both analog and digital methods.

### 4.1. Frequency Modulation with Analog Spread Spectrum Clock Generator

(A) Configuration and operation

**Figure 7:**
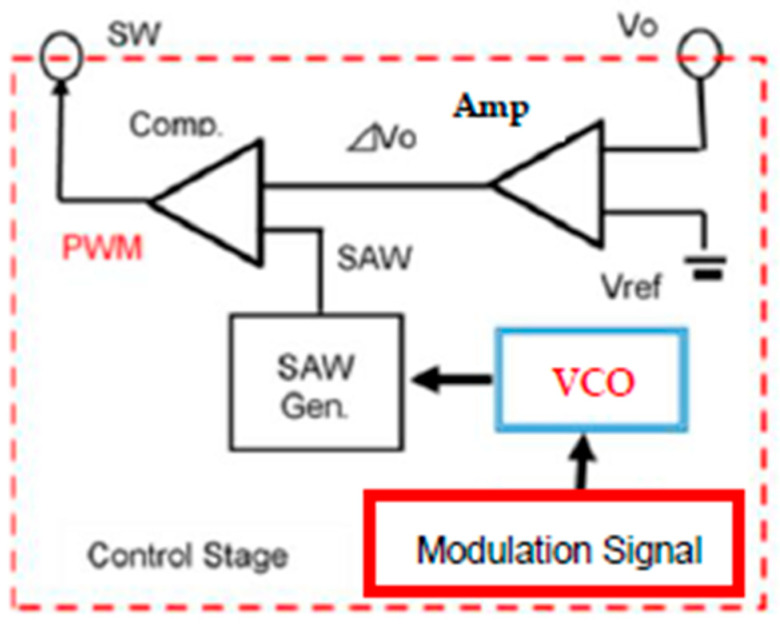
Frequency modulator in control stage.

**Figure 8:**
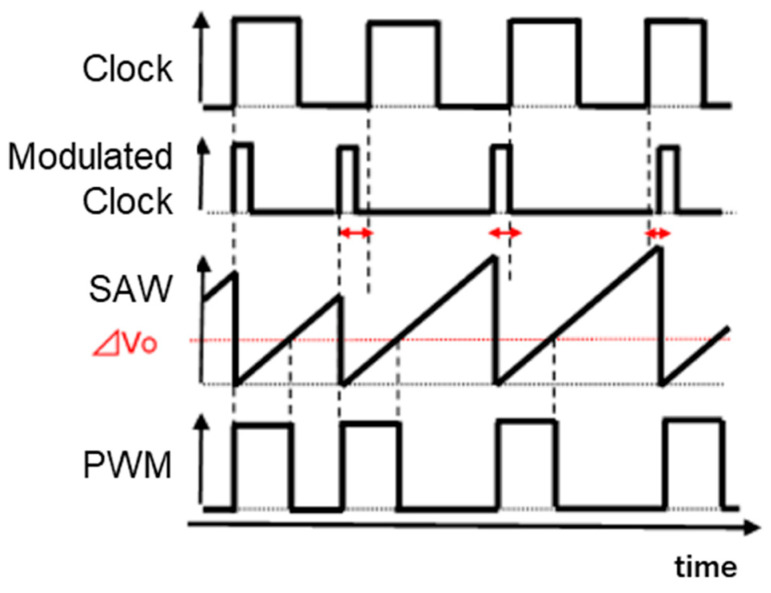
Signals for frequency modulation.

It is recognized that the clock frequency or phase modulation in switching converters spreads the power spectrum of the EMI noise from the PWM signal and decreases its peak level at the clock and harmonics frequencies. Figure 7 illustrates the clock frequency modulator, which replaces the control block in Figure 1 to implement the buck converter with a noise spread spectrum capability. Figure 8 shows its associated signals. There, the voltage-controlled oscillator (VCO) is incorporated for the modulation of the frequency or phase of the SAW signal.

**Figure 9:**
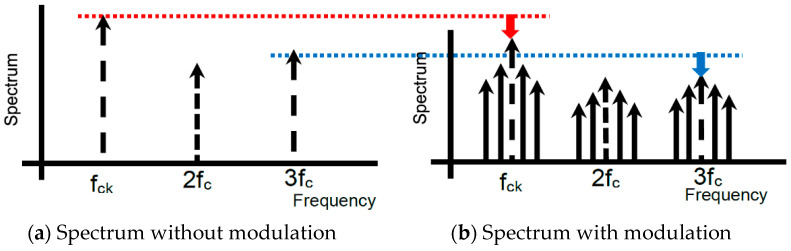
Spread spectrum of PWM pulse [36] @JTSS.

Figure 9a illustrates the spectrum of the PWM signal without clock modulation, whereas Figure 9b illustrates the one with modulation by two frequencies. The clock noise power spectrum is spread around the clock and harmonics frequencies. Several line spectra, indicated by the solid arrows, are discretely spread (marked by the dashed arrows); there, the peak power levels are reduced.

(B) Simulation results

**Figure 10:**
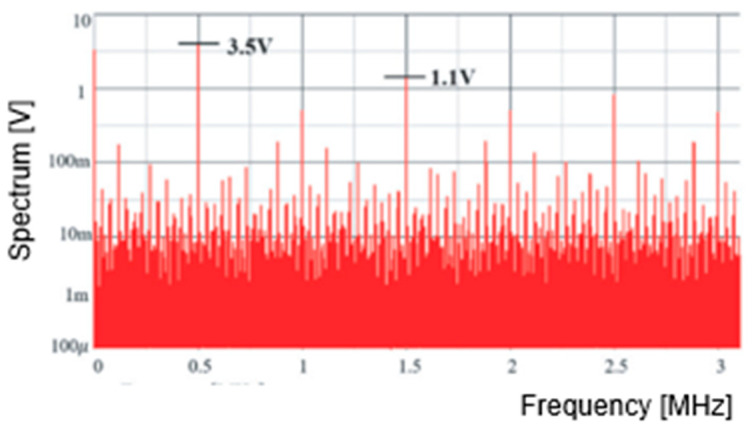
PWM spectrum without clock modulation.

**Figure 11:**
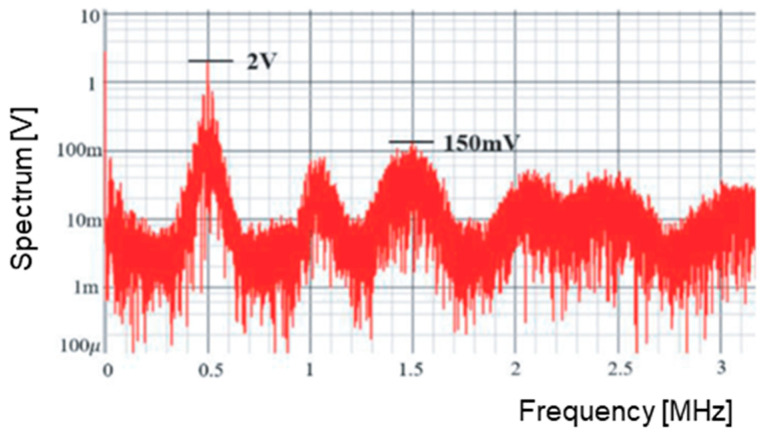
PWM spectrum with modulation.

Figure 10 illustrates the spectrum of the PWM pulse without clock modulation. At the clock frequency of 0.5 MHz, a line spectrum reaching 3.5 V is observed, along with many harmonics. For the clock noise reduction, its modulation is utilized by shaking its phase or frequency (Figure 7). Figure 11 displays the spectrum with modulation. Since the power at the clock and harmonics frequencies spread to other bands, their peak levels become lower, though the bottom level becomes higher.

### 4.2. Basic Digital Frequency Modulation with LFSR

(A) Configuration and generated pseudo analog signal

Pseudo-analog noise generation using a Phase Locked Loop (PLL) clock generator for modulation is reviewed.

**Figure 12:**
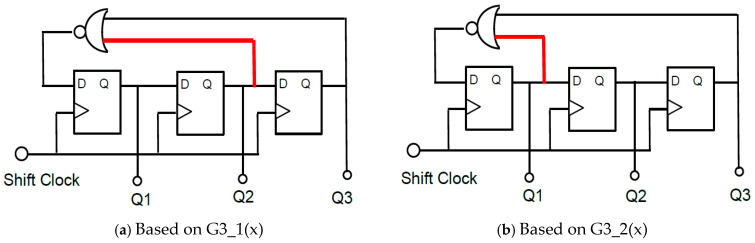
Three-bit LFSRs for two primitive polynomials [36] @JTSS.

Figure 12 illustrates the 3-bit linear feedback shift registers (LFSRs) or M-sequence circuits. Based on G3_1(x) in Equation (4), the outputs of the 3rd and 2nd bits are connected to the inputs of the Exclusive NOR (EX-NOR) gate, and its output is provided to the first DFF. The n-bit LFSR generates K (=2^n^ −1) levels, where n is the order of the primitive polynomial. For *n* = 3, K is 7, and there are two primitive polynomials, G3_1(x) in Equation (4) and G3_2(x) in Equation (5).

**Figure 13:**
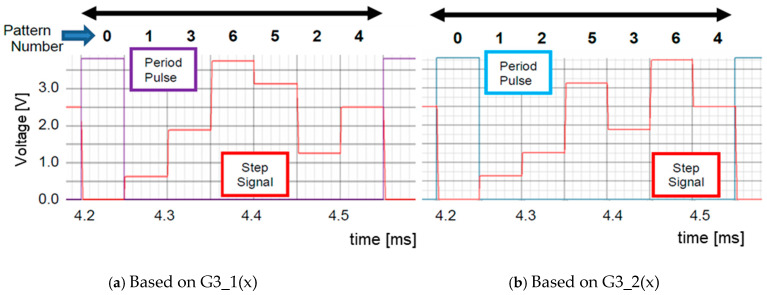
Waveforms of output levels with LFSRs [36] @JTSS.

**Figure 14:**
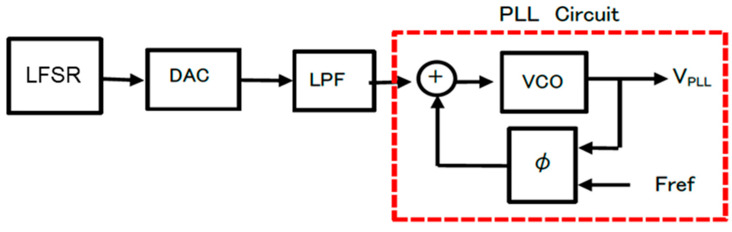
Configuration of the clock generator with analog modulation [36] @JTSS.

**Figure 15:**
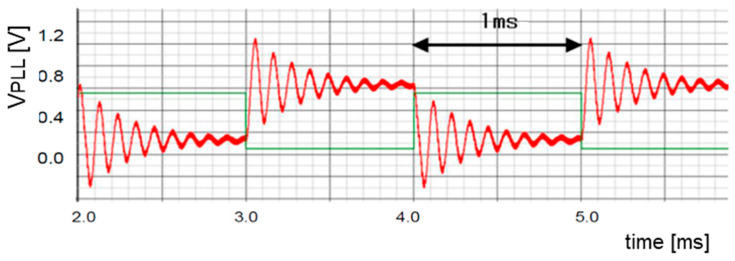
Step response of the PLL [36] @JTSS. Green: Step input. Red: Output.

Figure 13 illustrates the analog output levels obtained from (Q3, Q2, and Q1) by the LFSR in Figure 12. There are periodic patterns with seven levels: 0-1-3-6-5-2-4 or 0-1-2-5-3-6-4. These are transformed into a smooth signal as pseudo-analog noise. This pseudo analog noise is introduced into the PLL, and then its output is frequency-modulated (Figure 14). Also, the PLL does not lock to the input noise due to its sluggish step response characteristics (Figure 15).

G3_1(x) = x^3^ + x^2^ + 1 (4)

G3_2(x) = x^3^ + x + 1 (5)

(B) Simulation of clock modulation with pseudo analog noise

The clock generator with modulation by the pseudo analog noise is illustrated in Figure 14. The generated pulse train by the LFSR in Figure 12 is converted to the analog signal by the DAC and smoothed by the LPF, and it is supplied to the PLL. Figure 15 shows the simulated characteristics of the damped vibrated signal when the input signal is changed with some steps.

**Figure 16:**
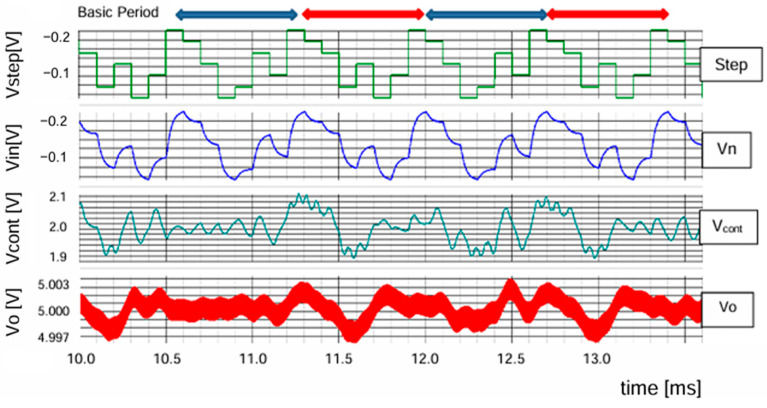
Signals associated with PLL circuit [36] @JTSS.

Figure 16 displays the pseudo analog noise (Vn: periodic), the VCO input signal (Vcont: non-periodic), and the output voltage ripple (⊿Vo: non-periodic) when an LFSR based on G3_1(x) is used. The clock frequency is 400 kHz, while the shift clock of the LFSR in Figure 12 is 10 kHz. Then, the periodic frequency of Vn is 1.43 kHz, and ⊿Vo is about 10 mVpp. Table 1 shows the simulation parameters, and SIMPLIS is used as simulator.

**Figure 17:**
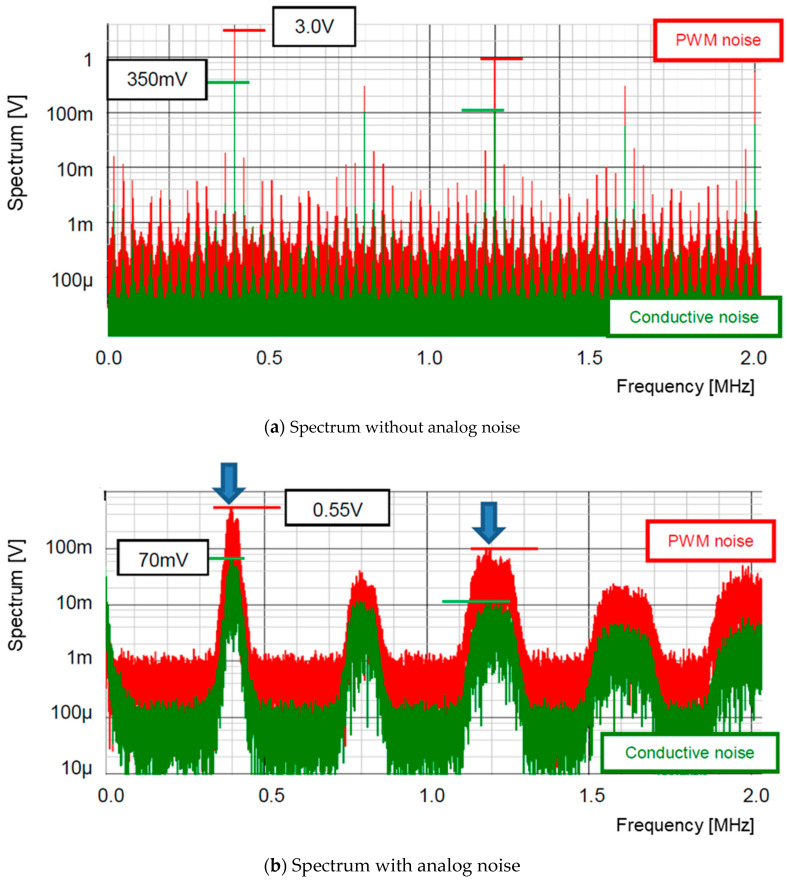
Spectrum of PWM and conductive noises [36] @JTSS.

Figure 17 displays the simulated spectra of the PWM signal (red) and the conducted noise (green). Figure 17a displays the spectra without the pseudo-analog noise (Np), whereas Figure 17b includes the ones with Np. The peak level at the clock frequency is reduced from 3.0 V to 0.55 V, and that at the 3rd harmonic is reduced from 1.0 V to 0.1 V, while that of the conductive noise is reduced from 350 mV to 70 mV.

### 4.3. Analog Noise Generators Using Bit Operation

(A) Expansion of analog pattern length using bit-inverse

We explain an analog noise generator using bit-exchange for an LFSR. The longer the period of the analog noise, the lower the peak level at the specific frequency, resulting in a broader sideband spectrum. The 3-bit system produces eight analog levels, and the number of permutations of the pattern levels is _7_P_7_ = 5040. Thus, a larger number of bits increases the number of permutations. For instance, inversing some of the bits from the LFSR output produces other pattern levels.

Additional patterns from the combination of these pattern levels are included. Equations (6)–(13) illustrate the results of the bit-inversed level streams of Equations (4) and (5). Usage of these streams extends the analog noise period to 8 times that of the original. The pattern length using the bit-inverse results in T1 = 8·2·7 = 16·To, where To is the original pattern length of 7.

        [Based on G3_1(x)]    :    [Based on G3_2(x)]

(1) Q1 Q2 Q3 :  0 - 1 - 3 - 6 - 5 - 2 - 4 -  :  0 - 1 - 2 - 5 - 3 - 6 - 4 - (6)

(7) $$(2)\;\bar{Q1}\;Q2\;Q3\;:\;\;1-0-2-7-4-3-5-\;\;:\;\;1-0-3-4-2-7-3-$$

(8) $$(3)\;Q1\;\bar{Q2}\;Q3\;:\;\;2-3-1-4-7-0-6-\;\;:\;\;2-3-0-7-1-4-6-$$

(9) $$(4)\bar{\;Q1}\;\bar{Q2}Q3\;:\;\;2-3-0-5-6-1-7-\;\;:\;\;3-2-1-6-0-5-7-$$

(10) $$(5)\;Q1\;Q2\;\bar{Q3}\;:\;\;4-5-7-2-1-6-0-\;\;:\;\;4-5-6-1-7-2-0-$$

(11) $$(6)\;\bar{Q1}\;Q2\;\bar{Q3\;}\;:\;\;5-4-6-3-0-7-1-\;\;:\;\;5-4-7-0-6-3-1-$$

(12) $$(7)\;Q1\;\bar{Q2}\;Q3\;:\;\;6-7-5-0-3-4-2-\;\;:\;\;6-7-4-3-5-0-2-$$

(13) $$(8)\;\bar{Q1}\;\bar{Q2}\;\bar{Q3}\;:\;\;7-6-4-1-2-5-3-\;\;:\;\;7-6-5-2-4-1-3-$$

(B) Simulation results of pattern generator with bit-inverse

**Figure 18:**
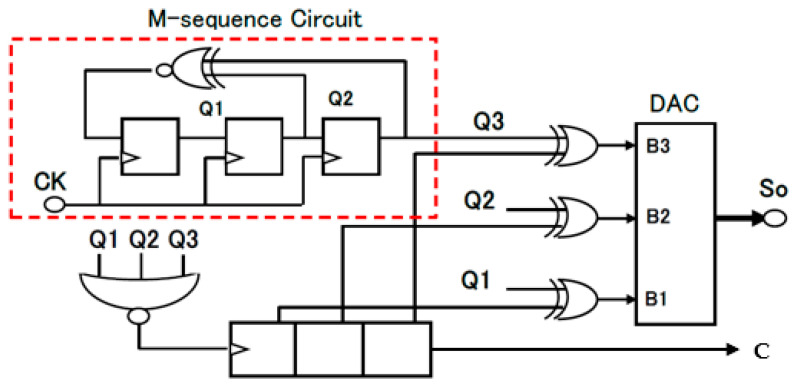
Pattern generator using bit-inverse [36] @JTSS.

**Figure 19:**
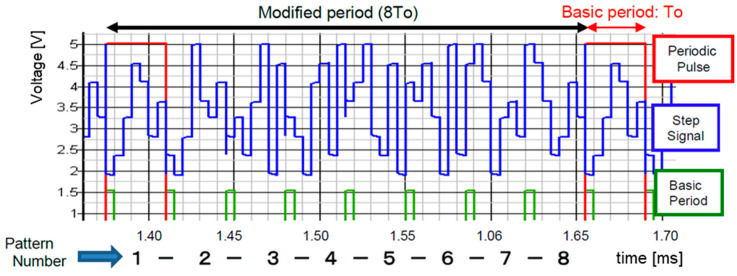
Expanded pattern length using bit-inverse [36] @JTSS.

Figure 18 illustrates the simulation circuit of the LFSR (M-sequence circuit) with bit-inverse based on G3_1(x). The bit-inverse uses three EX-NOR gates, in conjunction with a 3-bit binary counter, that is triggered once within a period of the LFSR counter. In Figure 18, the binary counter increments when all bits of the LFSR counter are zero, and CK is triggered when all the outputs of the binary counter are zero. The periodic length of the 3-bit DAC input (B3, B2, B1) extends to 8 times To (Figure 18 and Figure 19).

**Figure 20:**
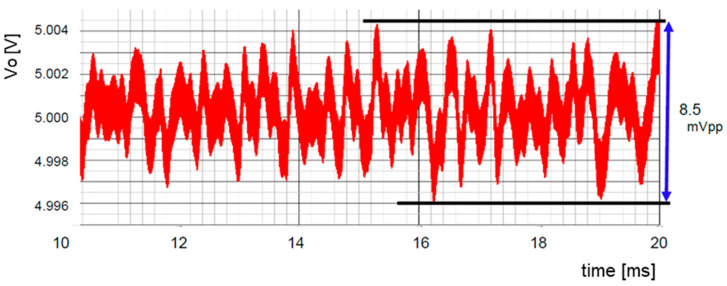
Output ripple of the buck converter using bit-inverse [36] @JTSS.

**Figure 21:**
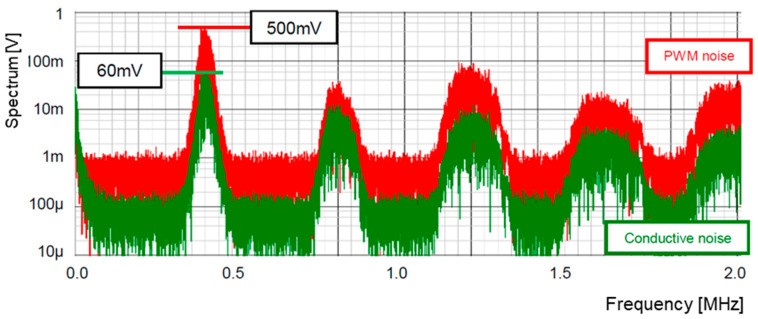
Spectrum of the buck converter output using bit-inverse [36] @JTSS.

Figure 20 displays the output voltage ripple (⊿Vo) of the converter using pseudo analog noise, while Figure 21 illustrates the spectrum of the PWM signal and the conductive noise using bit-inverse. The ripple voltage is 8.5 mVpp. The peak level at the clock frequency is reduced from 550 mV to 500 mV, and the level of the conductive noise is from 70 mV to 60 mV, compared to Figure 17b.

### 4.4. Pattern Generator Using Bit-Inverse and Bit-Exchange and Simulation Results

(A) Expansion of analog pattern length using bit-inverse and bit-exchange

The bit-exchange technique for expansion of the analog noise period is introduced. There are no identical bit streams in terms of Equations (6)–(13). The new pattern length becomes 6 times the one with only bit-inverse (T2 = 6·T1 = 96·To).

[Based on G3_1(x)]:[Based on G3_2(x)]

(1) Q1Q2Q3 : 0 - 1 - 3 - 6 - 5 - 2 - 4 - : 0 - 1 - 2 - 5 - 3 - 6 - 4 - [basic] (6)

(2) Q1Q3Q2 : 0 - 1 - 5 - 6 - 3 - 4 - 2 - : 0 - 1 - 3 - 5 - 2 - 4 - 6 - (14)

(3) Q2Q1Q3 : 0 - 2 - 3 - 5 - 6 - 1 - 4 - : 0 - 6 - 2 - 3 - 5 - 1 - 4 - (15)

(4) Q2Q3Q1 : 0 - 4 - 5 - 3 - 6 - 1 - 2 - : 0 - 4 - 3 - 2 - 5 - 1 - 6 -(16)

(5) Q3Q1Q2 : 0 - 2 - 6 - 5 - 3 - 4 - 1 - : 0 - 6 - 5 - 3 - 2 - 4 - 1 - (17)

(6) Q3Q2Q1 : 0 - 4 - 6 - 3 - 5 - 2 - 1 - : 0 - 4 - 5 - 2 - 3 - 6 - 1 - (18)

(B) Simulation results of pattern generator using bit-inverse and bit-exchange

**Figure 22:**
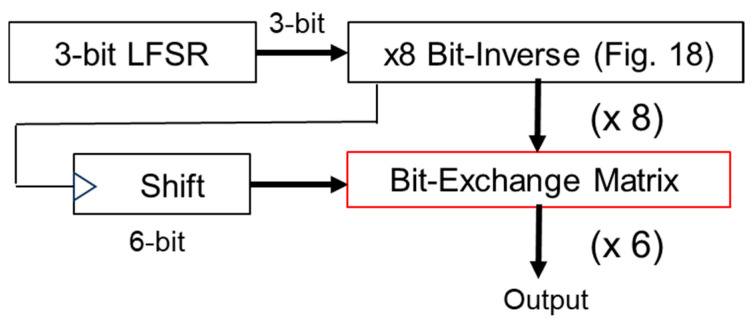
Pattern generator using bit-inverse and bit-exchange [36] @JTSS (Figure 18).

**Figure 23:**
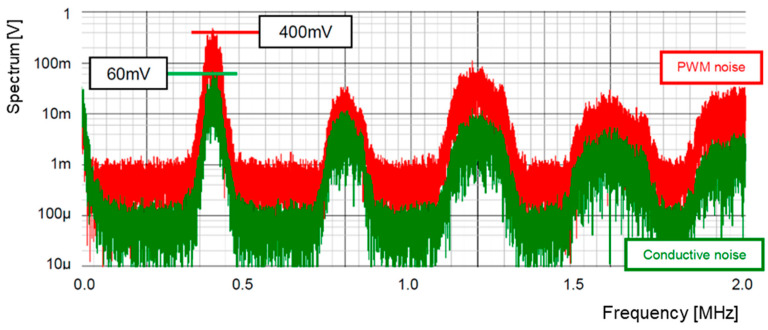
Spectrum with bit-inverse and bit-exchange [36] @JTSS.

Figure 22 illustrates the configuration of the LFSR using bit-inverse and bit-exchange. The bit-inverse circuit is shown in Figure 18, and the bit-exchange circuit is composed of a multiplexer array. At the 3-bit DAC output, the number of analog steps is 48 times that of the original pattern. Figure 23 illustrates its simulated spectra. The peak level of the PWM pulse noise is reduced from 500 mV to 400 mV (−17.5 dB), while the conductive noise remains 60 mV, compared to Figure 21.

### 4.5. Expansion of Number of Pseudo Analog Noise Generator Bits

(A) Expansion of number of bits

We found that it is not the number of bits for the LFSR that reduces EMI noise, but rather the ratio of the variation in pattern levels of the LFSR. There are two 4th-order primitive polynomials, and the bit level series are given in Equations (27) and (28), while the number of the pattern variations is To = 2^4^ − 1 = 15.

[4th-order]      G4_1(y) = y^4^ + y^3^ + 1 (19)

G4_2(y)= y^4^ + y + 1 (20)

[Pattern]      G4_1:0-1-3-7-14-13-11-6-12-9-2-5-10-4-8-(21)

G4_2:0-1-2-5-10-4-9-3-6-13-11-7-14-12-8-(22)

(B) Simulation results with 4th-order primitive polynomials

**Figure 24:**
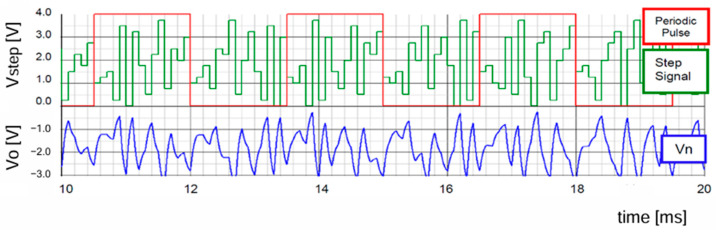
Signals of 4-bit LFSR with bit-inverse [36] @JTSS.

**Figure 25:**
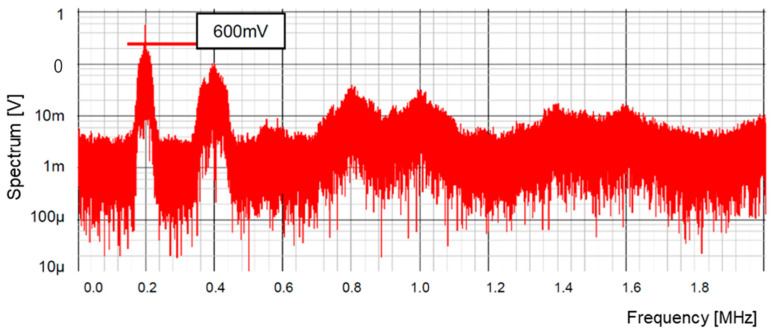
Spectrum of PWM pulse using 4-bit LFSR with bit-inverse [36] @JTSS.

Figure 24 illustrates the output step pattern from the 4-bit LFSR and the output of the LPF following the 4-bit DAC with the bit-inversed versions of Equations (19) and (20). The step pattern consists of non-periodic pulses, because the condition of the initial bit patterns in the LFSR varies based on the previous ones. Figure 25 illustrates the spectrum of the PWM pulse. The peak level is 600 mV, which matches the one with 3rd-order primitive polynomials.

There is little difference in the EMI reduction for the 3-bit and 4-bit LFSRs with the bit-inverse operation. Therefore, only the 3-bit LFSR is sufficient, and we see that fine frequency changes in modulation are not necessary.

## 5. Notch Band Select with Pulse Coding Control [36,37,38,39]

This section reviews our EMI noise spread spectrum techniques with notch band selection using pulse coding methods: pulse width coding (PWC), pulse cycle coding(PCC), and pulse width and phase coding (PWPC). The notches appear at frequencies determined by empirical equations. We demonstrate the relationships of the notch frequencies and the coded pulses in simulation. Also, their analytical formulae are shown.

### 5.1. Pulse Width Coding (PWC) Control

(A) Basic pulse coding control

**Figure 26:**
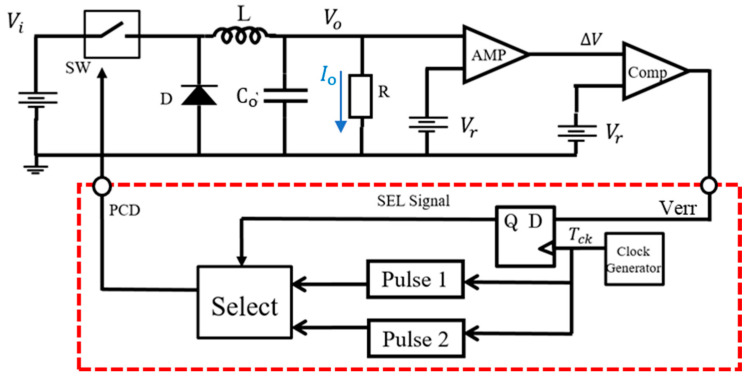
Switching converter with basic pulse coding control [37] @IEEE.

In the pulse coding control method, the switch is regulated by the Pulse-Coded Drive (PCD) signal, that is selected from two coded PWM pulses (Figure 26). Pulse 1 and Pulse 2 are generated using various coding methods with parameters for the pulse width, pulse phase, and their composite, and then, one is selected by SEL.

(B) Pulse Width Coding (PWC) control

**Figure 27:**
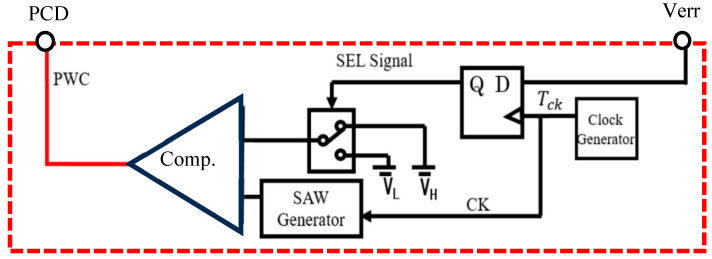
PWC control circuit.

**Figure 28:**
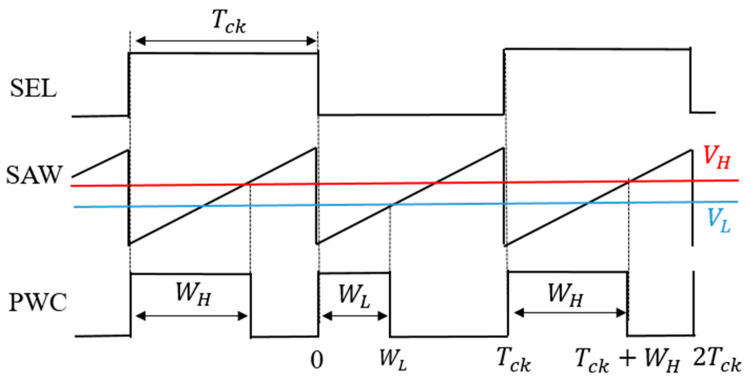
Signal waveforms for PWC control.

The PWC method discretely modulates the feedback pulse width. Figure 27 illustrates the PWC control circuit, and SEL is generated from the error voltage (Verr). Figure 28 illustrates the SEL, $V_{H}$, $V_{L}$, and PWC pulse ($W_{H}$ and $W_{L}$). When SEL is high, the multiplexer selects $V_{H}$, and the comparison with SAW generates $W_{H}$. When SEL is low, it selects $V_{L}$, and the comparison with SAW generates $W_{L}$. The output voltage is stably controlled by satisfying the following equation:

D_L_ < D_o_ (=V_o_/V_i_) < D_H_ (23)

Here, *D_L_ = W_L/_T_ck_*, and *D_H_ = W_H/_T_ck_* in Figure 28.

In the PWC control, the analog output voltage error is converted to a digital signal, and it modulates pulses. The converter output voltage is stabilized by appropriately switching these pulses. As a result, the noise spectrum is spread, and its notch is generated. Simulation shows that the notch frequency ($F_{n}$) is given as follows:

*F*_*n*_ = *N*/(*W*_*H*_ − *W*_*L*_) (24)

Hereafter, *N* is a positive integer throughout this paper. We observe that $F_{n}$ is determined by the difference of W_H_ and W_L_, and it is independent of the clock frequency. By adjusting W_H_ and W_L_, $F_{n}$ can be set arbitrarily.

(C) Simulation Results of PWC Control

The pulse-coded control regulates the converter output voltage using only two pulses, but without a sawtooth signal. The clock frequency is set to exceed 500 kHz to ensure precise control of the output voltage, and the other parameters are in Table 2.

**Figure 29:**
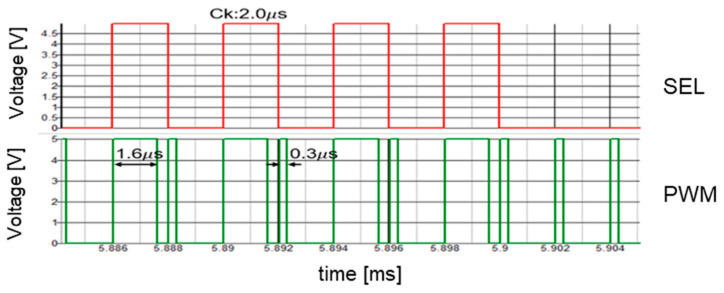
Signals of PWC control.

**Figure 30:**
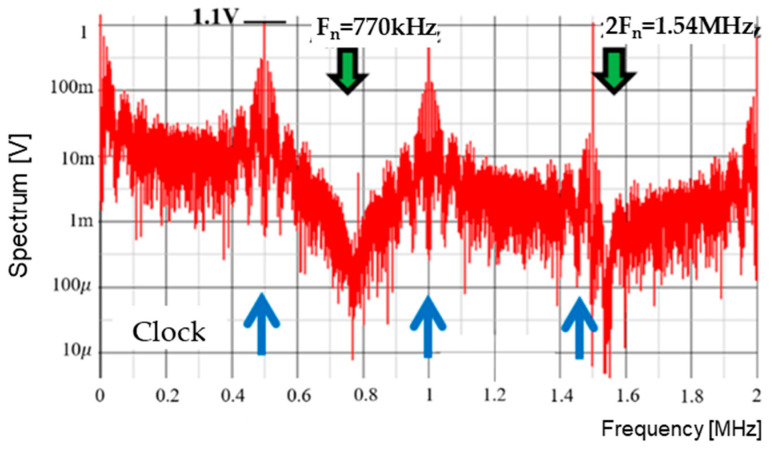
Spread spectrum of PCD signal with PWC control.

The SAW peak voltage is set to 12 V, $V_{H}$ is et to 9.6 V, and $V_{L}$ is set to 1.8 V. Comparisons of $V_{H}$ and $V_{L}$ with the SAW under $T_{ck}$ = 2 μs results in $W_{H}$ of 1.6 μs and $W_{L}$ of 0.3 μs, respectively. Figure 29 illustrates SEL and PWM signals, while Figure 30 displays the PWC signal spectrum. The up-arrows indicate the clock, its double, and its triple frequencies. A notch is observed at $F_{n}(=$770 kHz), corresponding to the theoretical frequency from Equation (24). Another notch is generated at 1.54 MHz, which corresponds to 2$F_{n}$. However, this notch is not very prominent due to the high-frequency noises from the clock. Comparing the peak level at the clock frequency in Figure 10 with that in Figure 30, it is suppressed from 3.5 V to 1.1 V. Also, notches are produced.

### 5.2. Pulse Phase Coding (PPC) Control

**Figure 31:**
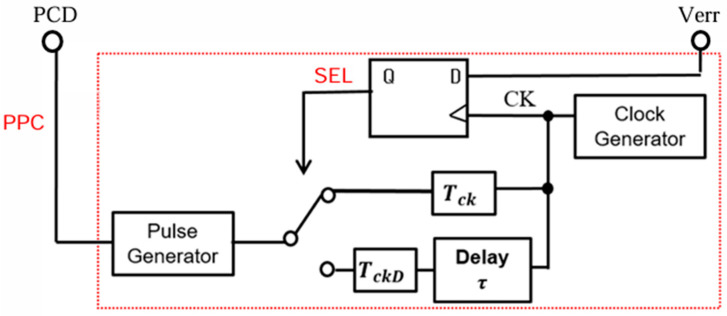
PPC control circuit.

**Figure 32:**
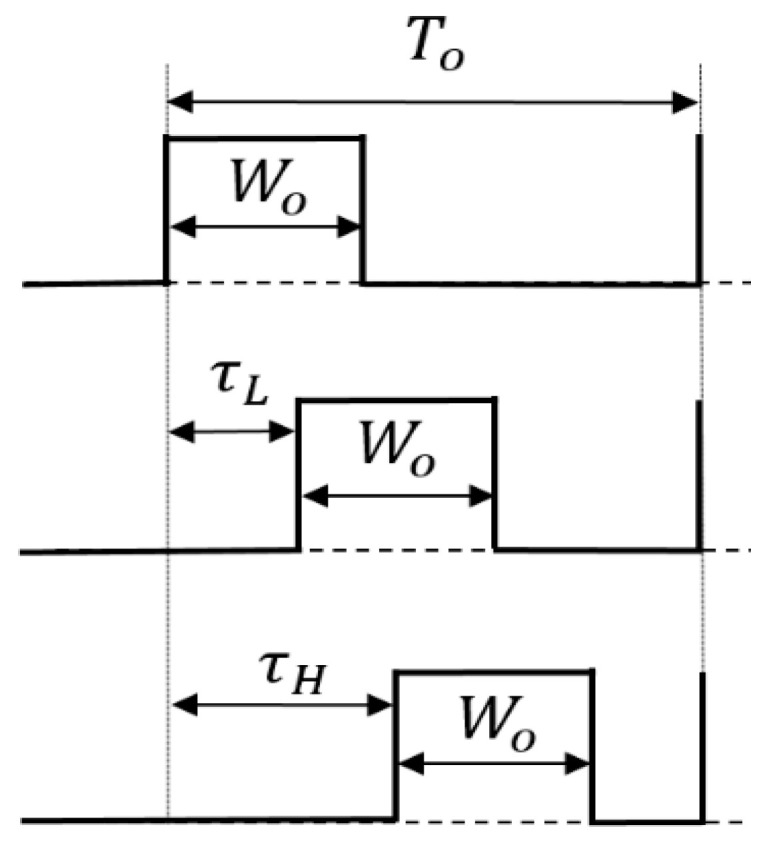
Signals of PPC control.

Pulse phase coding (PPC) control is realized with a delay circuit and a multiplexer (Figure 31). However, it does not alter D, and it is used with the PWC system. Parameters are used for the notch frequency based on the empirical formula (Equation (24)). Let τ represent the delay of pulse coding, with the longer delay represent as $\tau_{H}$ and the shorter one represented as $\tau_{L}$ (Figure 32). The notch characteristics are also obtained using the PCC method. In case of a pulse train with a clock cycle of $T_{o}$, the period *T*(*k*) of the *k*-th pulse is given by the following equation:

(25) $$T(k)=T_{o}+\{\tau (k)-\tau (k-1)\}$$

We see that in the PPC method, the notch is dependent on the previous pulse. Therefore, notches are unlikely produced because the coding cycle T(k) has 2^2^ patterns. To address this, the following two periodic patterns can be utilized if alternate high/low coding is applied to the phase coding:

*T*_*H*_ = *T* + {*τ*_*H*_ − *τ*_*L*_}, *T*_*L*_ = *T* − {*τ*_*H*_ − *τ*_*L*_} (26)

We obtain Equation (27) from Equations (24) and (26) as follows:

*F*_*np*_ = *N*/[2 (*τ*_*H*_ − *τ*_*L*_)] (27)

We see from Equation (27) that the notch characteristics are determined by twice the difference in the pulse phases.

### 5.3. Pulse Cycle Coding (PCC) Control

(A) PCC control circuit

**Figure 33:**
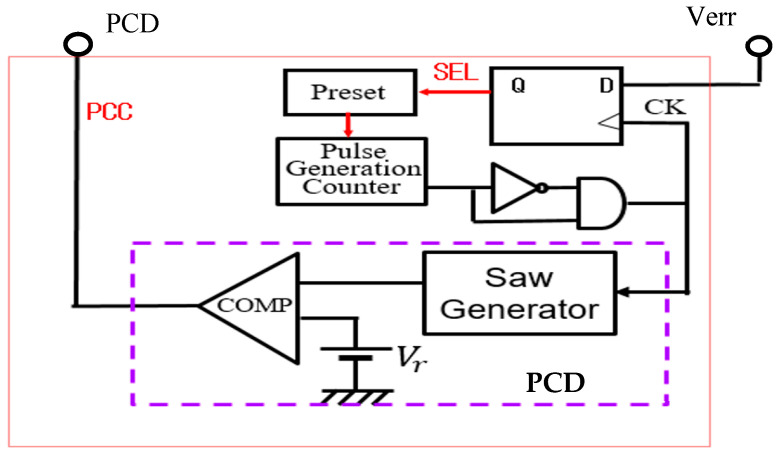
PCC control circuit.

**Figure 34:**
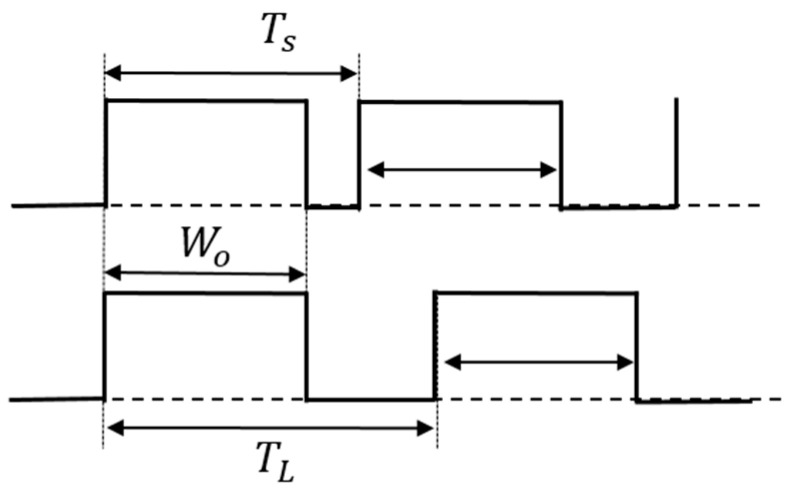
PCD pulse of PCC control.

**Figure 35:**
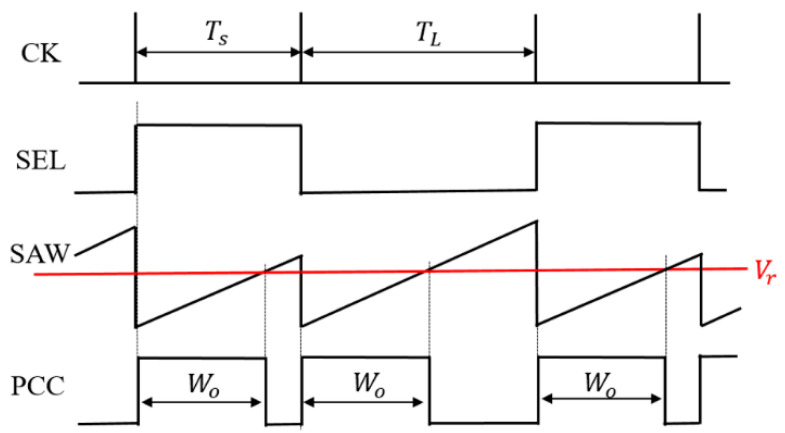
Main waveforms of PCC control.

In Figure 33, the duties of the two coded pulses differ as described by Equation (26). Consequently, the duty cycle changes by altering the pulse period (Figure 34). Figure 35 shows an example of two pulses using the PCC method. There, $W_{o}$ = 0.4 μs, $T_{s}$ = 0.5 μs, and $T_{L}$ = 2.0 μs. Consequently, $D_{H}$ = 0.8 and $D_{L}$ = 0.2. The notch frequency $F_{nc}$ in the spectrum of the PCD signal is given by

*F*_*nc*_ = *N*/(*T*_*L*_ − *T*_*S*_) (28)

Figure 33 shows the generator of these coded pulses. Pulses with different periods are generated from the pulse generation counter based on SEL (Figure 34). Here, $T_{L}$ and $T_{s}$ are defined as the pulse periods which are produced based on the SEL high and low states. By utilizing a differential circuit, a periodically modulated clock signal is generated. The generated sawtooth is compared with $V_{r}$, and the PCC pulse is produced. Figure 35 shows signal waveforms associated with the pulse coding in the PCC system. The clock cycle varies depending on SEL, and the PCC signal synchronized with that cycle is the output.

(B) Simulation Result with the PCC Control

**Figure 36:**
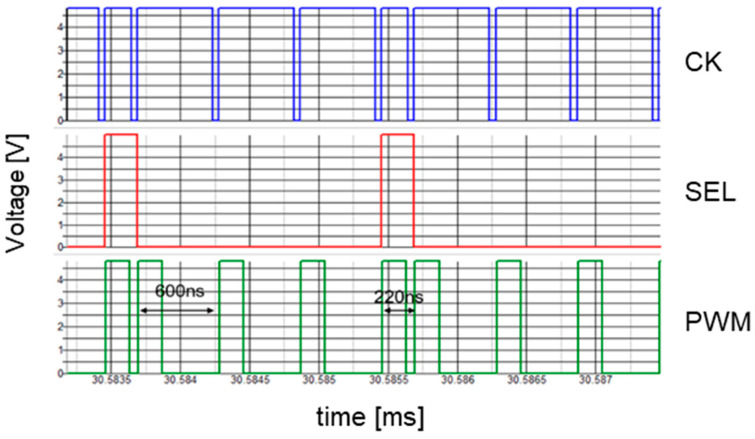
Simulated signals of PCC method.

**Figure 37:**
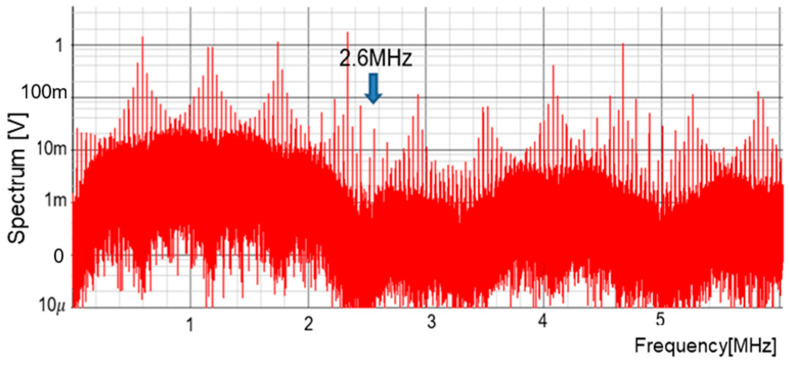
Spread spectrum of PCD signal with PCC control (without spread spectrum).

Figure 36 illustrates the main signals, and the pulse lengths of the PCC signal vary based on SEL. Figure 37 presents the simulated spectrum of the PCD signal with the PCC control. There, the pulse conditions are $T_{L}$ = 600 ns and $T_{s}$ = 220 ns, resulting in a notch frequency of $F_{nc}$ = 2.6 MHz, as calculated from Equation (28). However, in Figure 37, notches appear around $F_{nc}$ though they are not clearly visible. There are many line spectra because the spread spectrum technique is not utilized. The frequency position of the notch spectrum can be altered by the coded pulse frequencies or the switching converter parameters. Table 3 shows the simulation parameters.

### 5.4. Pulse Width and Phase Coding (PWPC) Control

(A) PWPC Method

**Figure 38:**
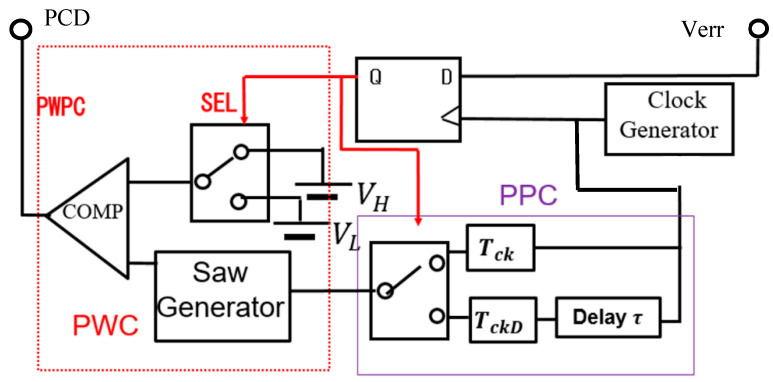
PWPC control circuit.

**Figure 39:**
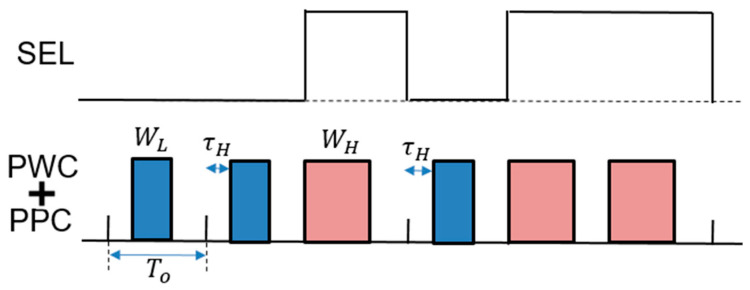
Signals of PWPC control in Figure 38. Light red: Narrow pulse. Light blue: Wide pulse.

The PWPC method is realized by incorporating a PPC circuit followed by the SAW generator for PWC (Figure 38). There, the frequency for a large notch is designed using Equations (27) and (28). Figure 39 illustrates the SEL and PWPC signals. When SEL is high, $W_{H}$ is selected, and when SEL is low, the shifted $W_{L}$ is selected. It is observed that the notch produced by the PWPC method is deeper compared to the PWC method.

(B) Simulation Results with PWPC Control

**Figure 40:**
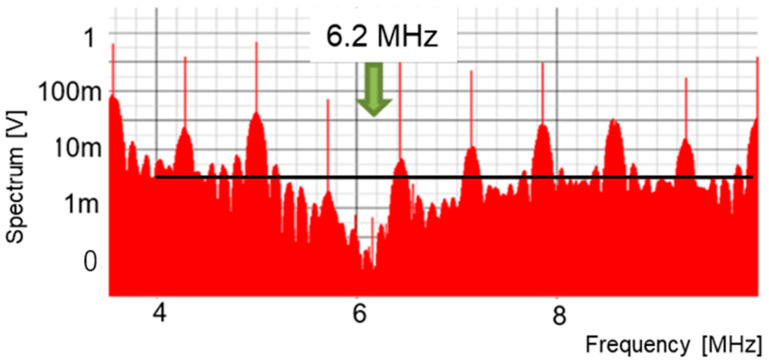
Spread spectrum of PCD signal with PWPC control.

In simulation, we set *T_o_* = 500 ns, *W_H_* = 320 ns, *W_L_* = 160 ns, τ_H_ = 80 ns, and τ_L_ = 0 ns to produce a large notch at 6.25 MHz. Figure 40 illustrates the simulated spectrum of the PWPC signal. The noise level at the notch frequency is less than −20 dB relative to the average level.

### 5.5. Derivation of Notch Frequency Using Fourier Transform

Now, we analyze various pulse coding methods and derive the formulae for their notch characteristics. We break down the analysis into four steps:

(1) Define the signal of the pulse coding method;
(2) Determine its Fourier transform;
(3) Take its absolute value to obtain their spectrum characteristics;
(4) Derive its zero point.

(A) Analysis of PWC Control

**Figure 41:**
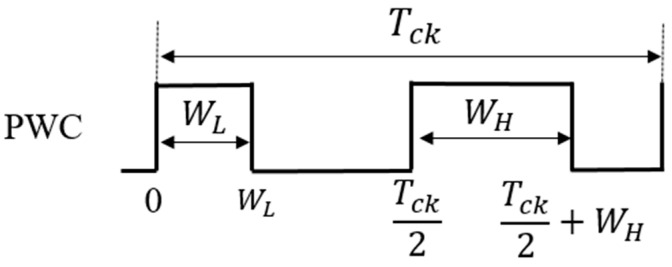
One-period two-pulse train of PWC signal.

First, we analyze the PWC control. One period of the PWC signal is defined as $T_{ck}$, which has two different pulse widths: $W_{L}$ and $W_{H}$ (Figure 41). The zero frequency of the PWC control spectrum is obtained as Equations (29)–(31), using Fourier transform on the pair of coding pulses.

(29) $$\begin{array}{c} F\left(\omega \right)=\int_{-\infty }^{\infty }f\left(t\right)e^{-j\omega t}dt\\ =\int_{0}^{W_{L}}f\left(t\right)e^{-j\omega t}dt+\int_{\frac{T_{ck}}{2}}^{\frac{T_{ck}}{2}+W_{H}}f\left(t\right)e^{-j\omega t}dt\\ =\frac{1}{\omega }\left[\sin\left(\omega W_{L}\right)-\sin\left(\;\omega W_{H}\right)+j\;\cos\left(\omega W_{L}\right)-j\;\cos\left(\;\omega W_{H}\right)\right] \end{array}$$

Then

$\omega^{2}F^{2}(\omega$) =$\;{\left[\sin\left(\omega W_{L}\right)-\sin\left(\;\omega W_{H}\right)+j\;\cos\left(\omega W_{L}\right)-j\;\cos\left(\;\omega W_{H}\right)\right]}^{2}$

(30) $$\omega^{2}|F\left(\omega \right){|}^{2}=4\;\sin^{2}\left(\frac{\;\omega }{2}(W_{H}-W_{L}\right))$$

Now we have the following:

(31) $$\begin{array}{c} \left|F\left(\omega \right)\right|=\frac{1}{\omega }2\;\sin(\;\frac{\;\omega W_{H}-\omega W_{L}}{2}\\ =\left(\;W_{H}-W_{L}\right)\frac{\sin\left(\frac{\;\omega (W_{H}-W_{L})}{2}\right)}{\frac{\;\omega (W_{H}-W_{L})}{2}}\\ =(W_{H}-W_{L})\;\sin c(\;\frac{\;\omega }{2}(W_{H}-W_{L})) \end{array}$$

We see that the PWC spectrum is expressed by a *sinc* function, which depends on the difference in pulse widths. The frequency at the zero point is obtained as follows:

(32) $$F_{notch}=N/\left(\;W_{H}-W_{L}\right)$$

Equation (32) shows that the notch characteristics correspond to the zero of the *sinc* function. Notice that the notch frequency is determined by the difference in pulse widths, and it is independent of the clock frequency.

**Figure 42:**
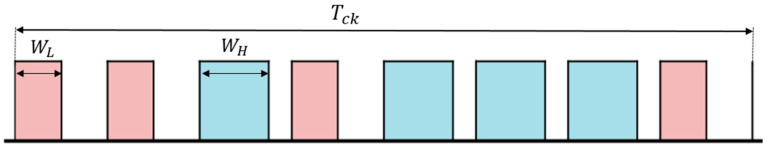
One-period eight-pulse train of PWC signal. Light red: Narrow pulse. Light blue: Wide pulse.

Next, calculate the spectrum characteristics of the eight rows of PWC pulses in Figure 42. Assume that the entire eight trains of pulses have a period $T_{ck}$, and the Fourier transform yields Equation (33).

(33) $$F\left(\omega \right)=-\frac{1}{j\omega }[\;\cos\left(\;\omega W_{H}\right)-j\;\sin\left(\omega W_{H}\right)+\cos\;\left(\omega W_{H}+\frac{\pi }{4}\right)-j\;\sin\;\left(\omega W_{H}+\frac{\pi }{4}\right)-\cos\left(\omega W_{L}\right)\;+j\;\sin\left(\omega W_{L}\right)-\cos\;\left(\omega W_{L}+\frac{\pi }{4}\right)+j\;\sin\;\left(\omega W_{L}+\frac{\pi }{4}\right)]$$

(34) $$\left|F\left(\omega \right)\right|=(W_{H}-W_{L})\;\sin c\left(\;\frac{\;\omega }{2}(W_{H}-W_{L}\right))\sqrt{6+4\;\cos\left(\frac{\pi }{4}\right)+4\;\cos\left(\frac{\pi }{2}\right)+4\;\cos\left(\;\frac{3\pi }{4}\right)}\;=\sqrt{14\;}(W_{H}-W_{L})\;\sin c(\frac{\;\omega }{2}(W_{H}-W_{L}))$$

The calculated result of the notch frequency based on Equation (33) is the same as that of Equation (32).

The notch characteristics depend solely on the difference of pulse widths, regardless of the arrangement and number of pulses. The frequency at the zero point is obtained by the following equation:

(35) $$F_{notch}=N/\left(W_{H}-W_{L}\right)$$

**Figure 43:**
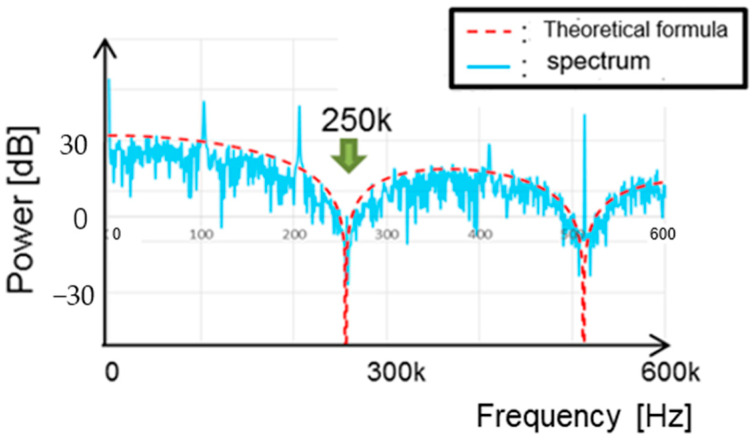
Comparison of theory and simulation.

Figure 43 illustrates a comparison between the *sinc* function and the spectrum of the PWC waveform with $W_{H}$ = 3 μs, $W_{L}$ = 7 μs, and $f_{notch}$ = 250 kHz. The envelopes of the spectrum in simulation match the theoretical result (Equation (35)).

(B) Analysis of PPC and PCC controls

**Figure 44:**
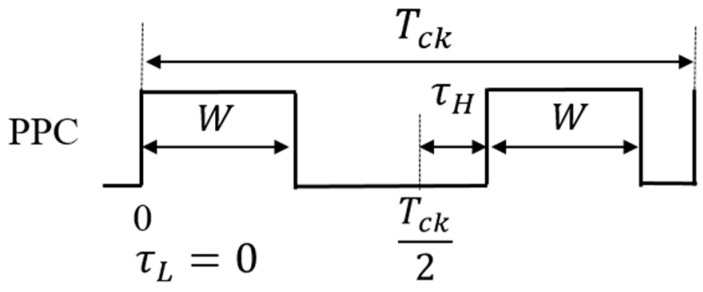
One-period two-pulse trains of PPC signal.

We analyze the pulse position coding (PPC) method. As illustrated in Figure 44, we define the PPC signal in one period as $T_{ck}$, which consists of two different phase pulse coding signals ($\tau_{H}$ and $\tau_{L}$). The frequency characteristics of the PPC control are obtained by Fourier transform on the pair of coding pulses (Equation (36)).

(36) $$F_{p}(\omega )=\int_{-\infty }^{\infty }f\left(t\right)e^{-j\omega t}dt\;=\int_{\tau_{L}}^{\tau_{L}+W}e^{-j\omega t}dt+\int_{\frac{T_{ck}}{2}+\tau_{H}}^{\frac{T_{ck}}{2}+\tau_{H}+W}e^{-j\omega t}dt\;=\frac{1}{\omega }[j\cos\left(\omega (\tau_{L}-\tau_{H}\right)+\sin\left(\omega (\tau_{L}-\tau_{H}\right))-j\;\cos\left(\omega \left(\tau_{L}-\tau_{H}-W\right)\right)\;-\sin\left(\omega \left(\tau_{L}-\tau_{H}-W\right)\right)-j\;\cos\left(\omega (\tau_{H}-\tau_{L}\right))-\cos\left(\omega (\tau_{H}-\tau_{L}\right))\;+j\;\cos\left(\omega \left(\tau_{H}-\tau_{L}-W\right)\right)+\sin\left(\omega \left(\tau_{L}-\tau_{H}-W\right)\right)$$

By taking the absolute value, Equation (37) is derived, which shows the frequency at the zero point as Equation (38):

(37) $$\left|F_{p}\left(\omega \right)|=2|\tau_{H}-\tau_{L}|\sin c\{\omega |\tau_{H}-\tau_{L}|\}||\sin(W\;\frac{\omega }{2})\right|$$

(38) $$F_{notch1}=N/[2|\tau_{H}-\tau_{L}|],\;\;F_{notch1}=N/W\;$$

According to Equations (37) and (38), the PPC method relies on two *sinc* functions, each exhibiting distinct notch characteristics. This method relies on the coding phase and the pulse width. Here, Equation (38) represents the theoretical equation of the PPC method when alternating coding is employed.

**Figure 45:**
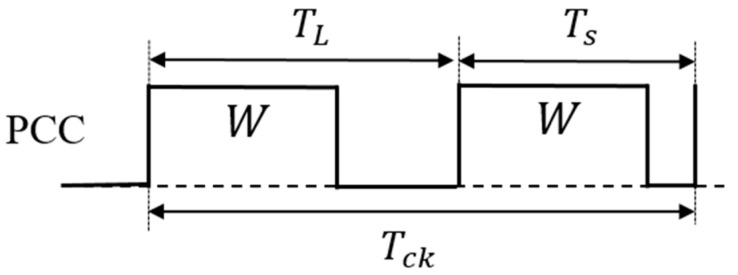
One-period two-pulse train of PCC signal.

Next, we analyze the PCC method. We define the PCC signal in one period as $T_{ck}$, which consists of two different cycle coding signals: $T_{L}$ and $T_{s}$ (Figure 45). The theoretical frequency of the PCC control is obtained as Equation (39) by Fourier transform on the pair of coding pulses.

(39) $$F_{c}(\omega )=\int_{-\infty }^{\infty }f\left(t\right)e^{-j\omega t}dt\;=\int_{0}^{T_{L}-W}e^{-j\omega t}dt+\int_{T_{L}}^{T_{S}-W}e^{-j\omega t}dt\;=\frac{1}{\omega }[j\cos\left(\omega T_{S}\right)+\sin\left(\omega T_{S}\right)-j\;\cos\left(\omega \left(T_{S}-W\right)\right)-\sin\left(\omega \left(T_{S}-W\right)\right)\;-j\;\cos\left(\omega T_{L}\right)-\sin\left(\omega T_{L}\right)+j\;\cos\left(\omega \left(T_{L}-W\right)\right)+\sin\left(\omega \left(T_{L}-W\right)\right)$$

(40) $$\;\;\;\;|F_{c}\left(\omega \right)|=2|T_{L}-T_{s}||\sin c\{\left(T_{L}-T_{s}\right)\frac{\omega }{2}\;\}||\sin(W\;\frac{\omega }{2})|$$

This equation shows that the notch characteristic depends on both the coding period and the pulse width, as in the PPC method.

(C) Analysis of PWPC method

**Figure 46:**
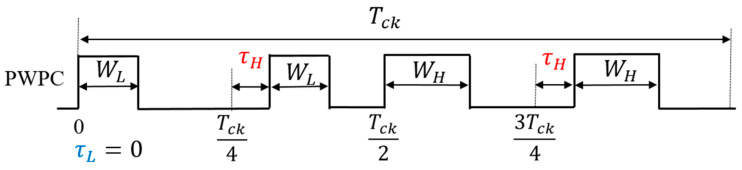
One-period four-pulse-train of PWPC signal.

Next, we analyze the pulse width and phase coding (PWPC) method, which encodes both the pulse width and phase. We define the PWPC signal in one period as $T_{ck}$, which represents two coding signals (Figure 46). The frequency characteristics of the PWPC control are derived as Equations (41) and (42).

(41) $$\;\;\;\;\;F_{c}\left(\omega \right)=\int_{-\infty }^{\infty }f\left(t\right)e^{-j\omega t}dt\;=\int_{\tau_{L}}^{\tau_{L}+W_{L}}e^{-j\omega t}dt+\int_{\frac{T_{ck}}{4}+\tau_{H}}^{\frac{T_{ck}}{4}+\tau_{H}+W_{L}}e^{-j\omega t}dt+\int_{\frac{T_{ck}}{2}+\tau_{L}}^{\frac{T_{ck}}{2}+W_{H}}e^{-j\omega t}dt+\int_{\frac{3\;T_{ck}}{4}+\tau_{H}}^{\frac{3\;T_{ck}}{4}+\tau_{H}+W_{H}}e^{-j\omega t}dt$$

(42) $$|F_{wc}(\omega )|=2|\tau_{H}-\tau_{L}||\sin c\text{\{}2|\tau_{H}-\tau_{L}|\frac{\omega }{2}\text{\}}||\sin(\left(W_{H}-W_{L}\right)\frac{\omega }{2}$$

In the PWPC, a *sinc* function with the pulse width and phase is used for representation, and two notch characteristics are generated. Further, if the notch characteristics are set to overlap with $2\left|\tau_{H}-\tau_{L}\right|=|W_{H}-W_{L}$∣, they become as follows:

(43) $$\;|F_{wc}\left(\omega \right)|=\frac{\sin^{2}(\frac{\omega }{2}(W_{H}-W_{L}\;))}{\frac{\omega }{2}}$$

**Figure 47:**
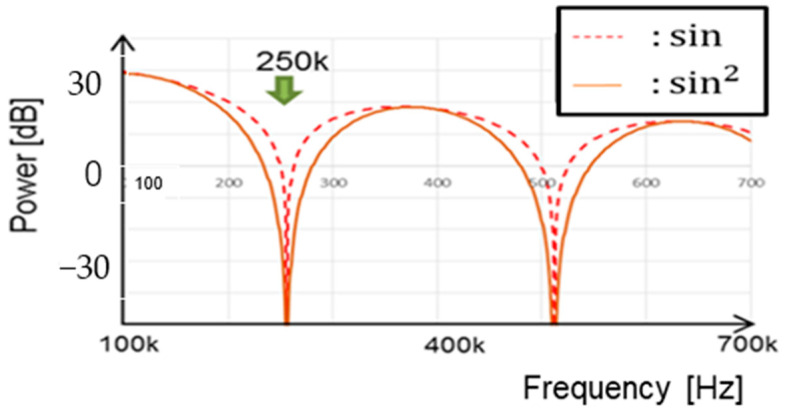
Comparison of notch characteristics with PWC and PWPC methods.

Figure 47 provides a comparison of the notch characteristics in Equations (43) and (31). The notch around the zero point at 250 kHz in Equation (43) is broader than in Equation (38). The composite coding method increases both the notch width and depth compared to the PWC or PPC method.

## 6. Automatic Notch Generation [36,37,38,39]

This section describes the generation method of $W_{H}$ and $W_{L}$ for automatic notch generation in PWC control and PWPC control.

### 6.1. Automatic Notch Generation Using PWC Control

(A) Design of Relationship Among $F_{n}$, $F_{ck},\;$and $T_{in}$

**Figure 48:**
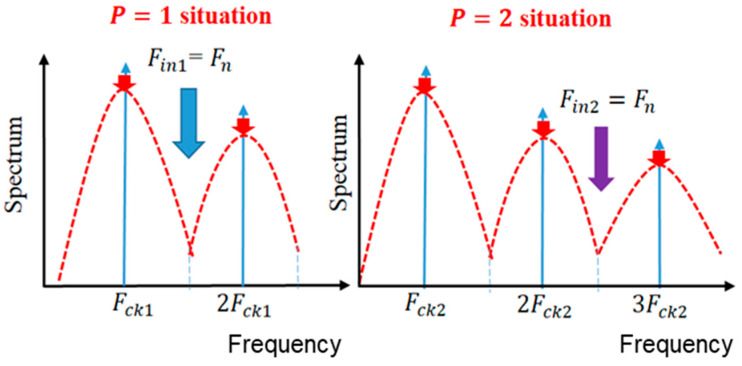
Positions of $F_{n}$ [27] @IEICE.

Our design is the generation of the notch frequency $F_{n}$ so that it lies between $F_{ck}$ and 2$F_{ck}$, and it matches the received signal frequency (Figure 48). $F_{in}$ is provided to match the receiving frequency, and the relationship among $F_{in}$, $F_{n}$, and $F_{ck}$ is given by Equation (44). Here, *P* is a positive integer. Then, the relationship of $T_{in}$ (=$1/F_{in})\;$and $T_{ck}\left(=1/F_{ck}\right)\;$is given by Equation (45).

(44) $$F_{in}=\left(P+0.5\right)F_{ck}$$

(45) $$T_{ck}=\left(P+0.5\right)T_{in}$$

We see from Equation (44) that in case of *P* = 1, $F_{n}$ is set between $F_{ck1}$ and 2$F_{ck1}$, and it is equal to $F_{in1}$. In the case of P = 2, $F_{n}$ is produced between $2F_{ck2}$ and 3$F_{ck2}$, and $F_{n}=F_{in2}$ (Figure 48).

**Figure 49:**
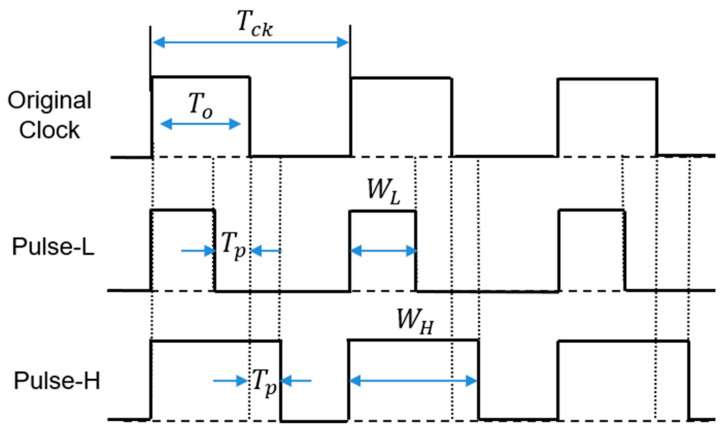
Timing of pulse-H and pulse-L [38] @IEEE.

Conversely, the duty ratio *D* of the PWC signal is represented by Equations (46)–(48). Further, the original clock signal in Figure 49 represents the PWC signal that is not encoded, with a pulse width of $T_{o}$. It also corresponds to Equation (46), where $D_{o}$ is set to 0.5. Pulse-H and pulse-L are generated based on $T_{o}$ (Figure 49), and this corresponds to Equation (47). Here, $T_{p}$ represents the difference of $W_{H}$ and $T_{o}$, or of $T_{o}$ and $W_{L}$. $T_{n}$ $\left(=1/F_{n}\right)\;$is determined by the difference of $W_{H}$ and $W_{L}$. Also, $W_{H}$, $W_{L}$, and $T_{o}$ must satisfy the relationship given in Equation (48) to ensure a stable output voltage, $V_{o}$. Here, 2 $T_{p}$ = $T_{n}$, and it is the difference of $W_{H}$ and $W_{L}$.

(46) $$T_{o}=D_{o}\times T_{ck}=\frac{V_{o}}{V_{i}}\times \;T_{ck}=0.5\;T_{ck}$$

(47) $$W_{H}=T_{o}+T_{p},\;W_{L}=T_{o}-T_{p}$$

(48) $$T_{n}=W_{H}-W_{L}=2\;T_{p}$$

(B) Automatic Notch Generation from Clock

**Figure 50:**
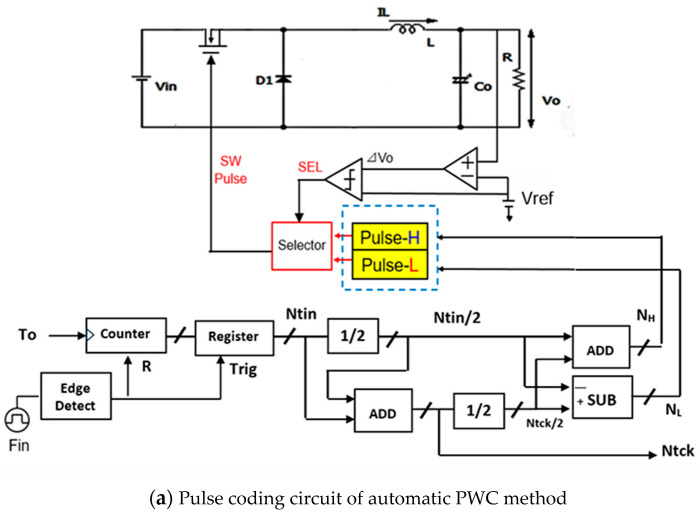

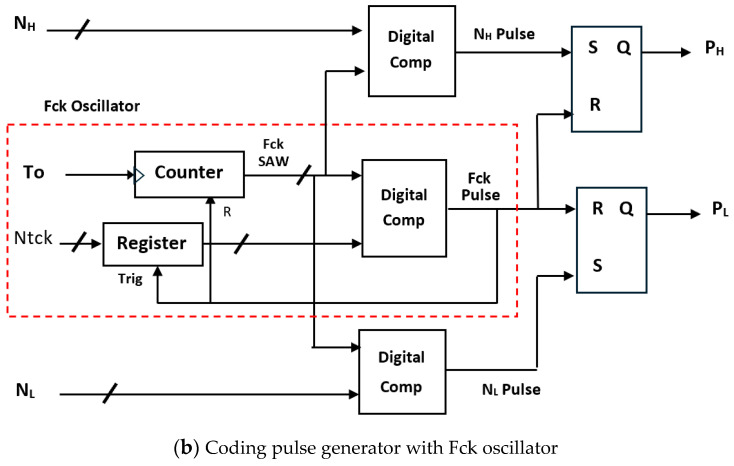
Pulse coding circuit of automatic PWC method for P=1 [38] @IEEE.

$T_{ck}$ is generated by $T_{in}$ as shown in Equation (45). For *P* = 1, the notch frequency is produced between $F_{ck}$ and 2$F_{ck}$ from $T_{in}$ (See Figure 48). $T_{ck}$ is shown in Equation (49), which can be realized with a shifter and an adder. Figure 50a,b show the automatic PWC pulse coding circuit based on Equations (46)–(48) for $D_{o}=0.5$, where $W_{H}=0.5T_{ck}+0.5T_{in},\;$and $W_{L}=0.5T_{ck}-0.5T_{in}$. Figure 50a shows the whole block diagram, while Figure 50b shows the detailed block diagram of pulse-H and pulse-L generation from $N_{H},N_{L}$, and $N_{tclk}$. Then, in the case of *P* = *N*, $F_{n}$ is produced between *N*$F_{ck}$ and (*N+*1)$F_{ck}$ with $T_{ck}$ as shown in Equation (50).

(49) $$T_{ck}=\left(1+0.5\right)T_{in}\to \;T_{ck}=1.5\;T_{in}$$

(50) $$T_{ck}=\left(N+0.5\right)T_{in}$$

**Figure 51:**
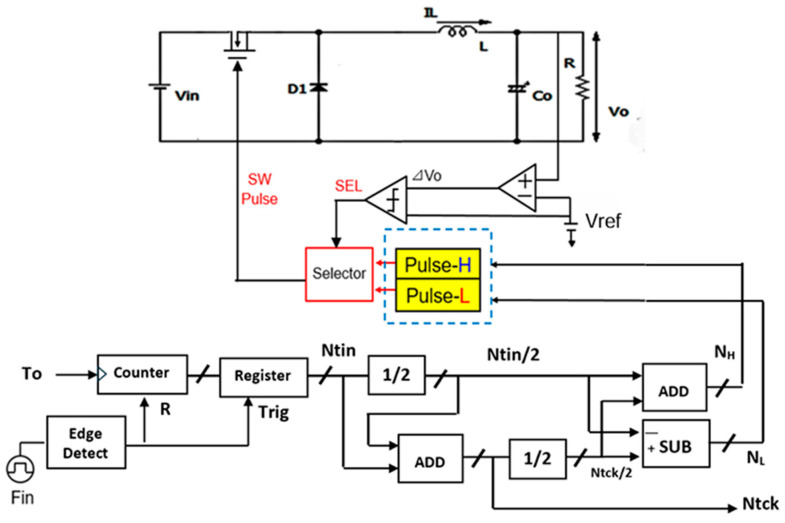
Pulse coding circuit of automatic PWC method for P=N.

Figure 51 illustrates the automatic PWC method for *P* = *N*. For instance, in the case of *N* = 2, $F_{in}$ is set to 1.25 MHz, $F_{ck}$ is 500 kHz, and $F_{n}$ appears at 1.25 MHz, which falls between 2 $F_{ck}$ and 3 $F_{ck}$.

(C) Simulation Results with Automatic Notch Generation

**Figure 52:**
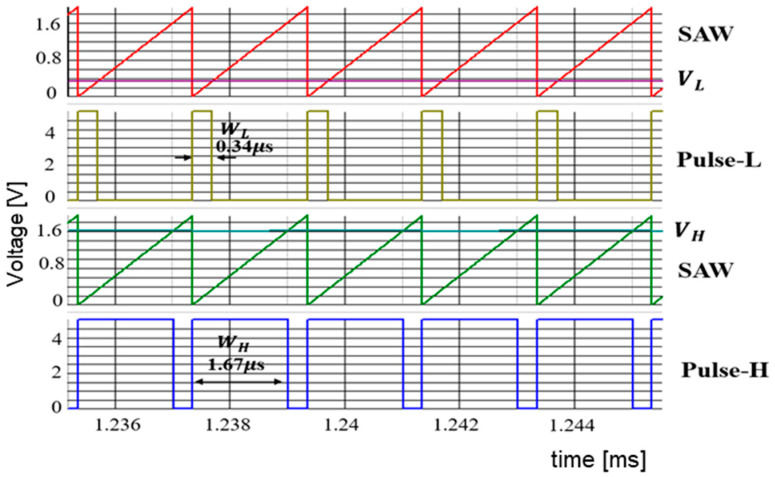
Simulated waveforms for P=1.

**Figure 53:**
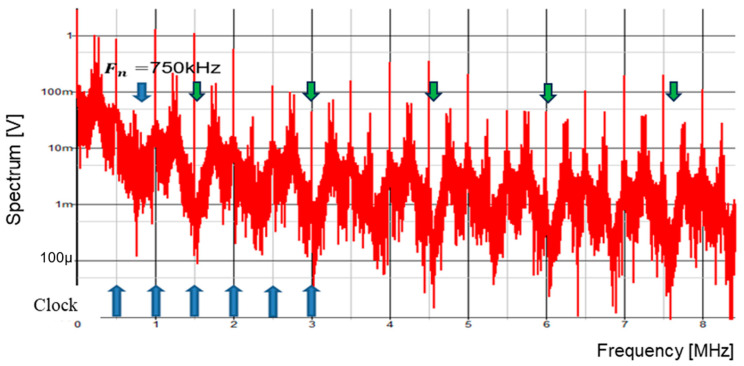
Simulated spectrum of PWM signal without spread spectrum (P = 1).

A digital circuit is used for coding pulse notch generation for P=1 (Figure 50). Figure 52 displays the waveforms of pulse-H and pulse-L for $F_{in}$ = 750 kHz. The sawtooth period $T_{ck}$ is automatically set to 2 μs. By comparing $V_{L}$ and $V_{H}$, pulse-L and pulse-H with $W_{L}=0.34\;\mu s$ and $W_{H}=1.67\;\mu s$ are generated automatically. According to Equation (23), $F_{n}$ is 750 kHz, and the spectrum of the PWM signal is shown in Figure 53. The notch is at 750 kHz, which corresponds to $F_{in}$, and there the bottom level is 1 mV. However, there is the line spectrum at $F_{in}$ (=500 kHz) with an amplitude of 900 mV, and multiple harmonics spectra are present. Thus, frequency modulation is considered for spread spectrum in the coding pulse notch generator.

**Figure 54:**
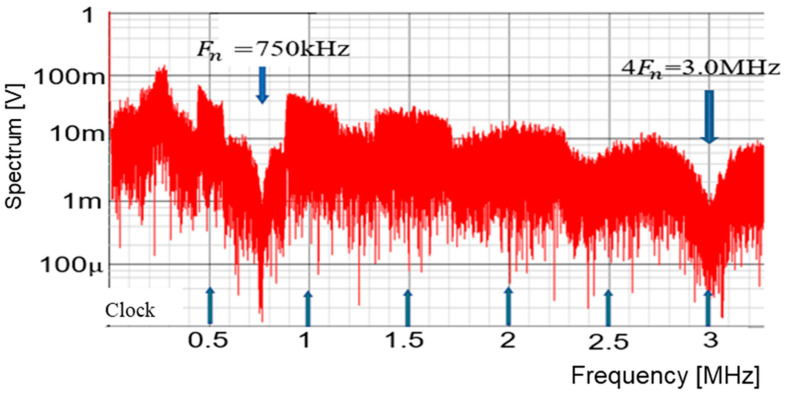
Simulated spectrum with spread spectrum for P=1.

The frequency modulation of $F_{ck}$ is used for EMI reduction as described in Section 5.1, and the spectrum of the PWM signal is displayed in Figure 54. The notch is clearly observed at 750 kHz ($=F_{in})$. The bottom level of the notch frequency is 1 mV, while the spectrum at $F_{ck}$ (=500 kHz) is 20 mV. Notice that another notch appears also at 4$F_{in}$ in simulation. In principle, a 3 MHz frequency is equal to 6$F_{ck}\;(=4F_{in}$). Since the clock and the input signal overlap, the notch is not expected to appear at 4$F_{in}$. The theoretical reason for the notch at 4$F_{in}\;$remains unknown and will be addressed in future work.

**Figure 55:**
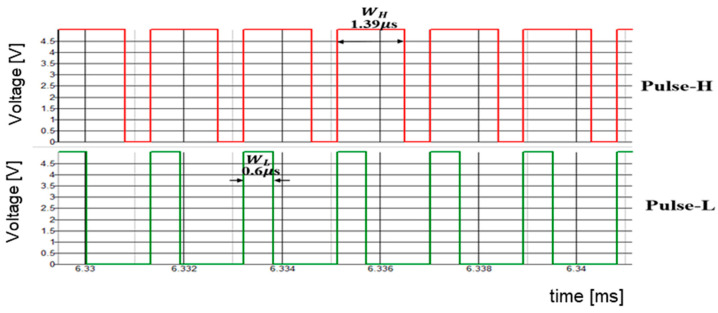
Simulated waveforms for P=2.

**Figure 56:**
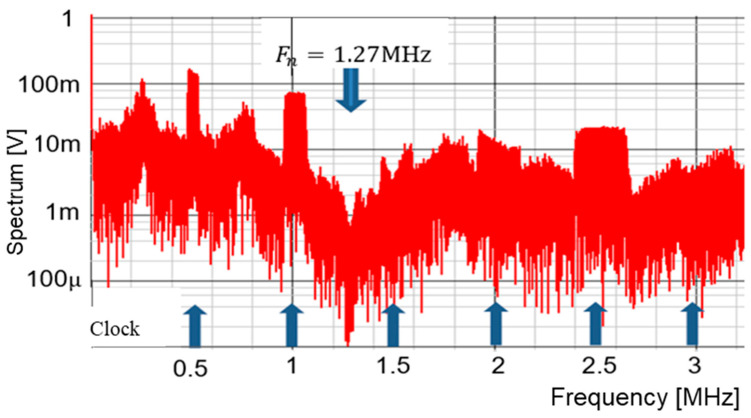
Simulated spectrum with spread spectrum for P=2.

Next, we discuss the case for P = 2. Figure 55 displays the simulated waveforms of pulse-H and pulse-L for $F_{in}$ = 1250 kHz, and we observe $W_{H}=1.39\;\mu s$ and $W_{L}=0.6\;\mu s$. The expected $F_{n}$ is 1250 kHz based on Equation (24). The spectrum of the PWM signal is displayed in Figure 56. The notch is observed at 1270 kHz, which is equal to $F_{in}$ and falls between 2$F_{ck}$ and 3$F_{ck}$.

**Figure 57:**
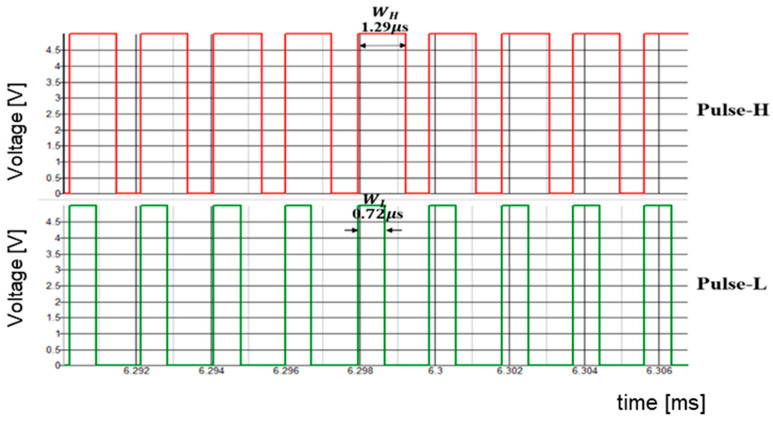
Simulated waveforms for P=3.

**Figure 58:**
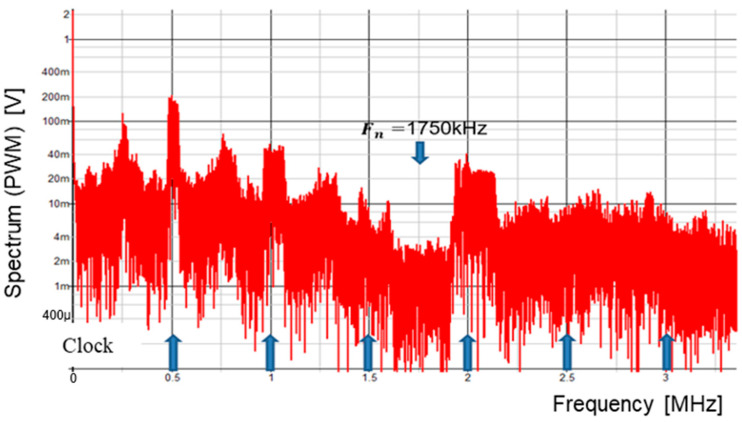
Simulated spectrum with spread spectrum for P=3.

Then, we consider the case for P = 3. Figure 57 displays the simulated waveforms of pulse-H and pulse-L for $F_{in}$ = 1750 kHz. Also, $W_{H}=1.29\;\mu s$ and $W_{L}=0.72\;\mu s$. Based on Equation (24), $F_{n}$ is 1750 kHz. The spectrum of the PWM signal is displayed in Figure 58. The notch is observed at 1750 kHz, which corresponds to $F_{in}$ and lies between 3 $F_{ck}$ and 4 $F_{ck}$.

(D) Automatic Setting of Notch Frequency from Input Frequency

**Figure 59:**
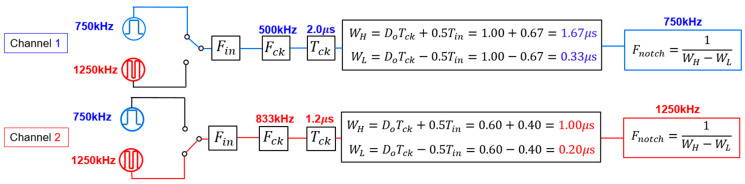
Block diagram of change from channel 1 to channel 2.

**Figure 60:**
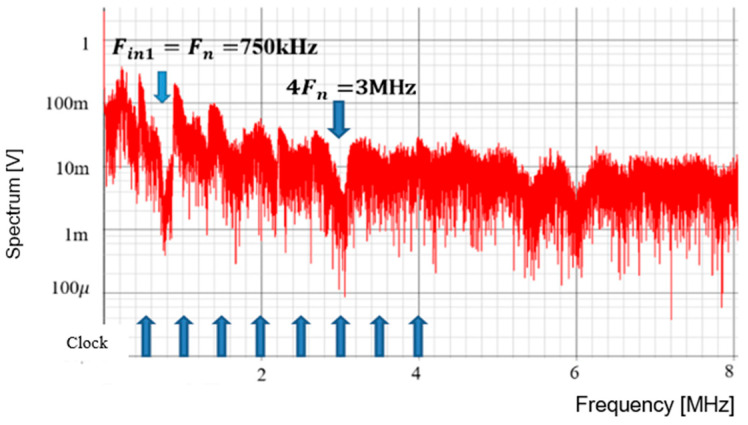
Simulated spectrum for$\;F_{in1}=750\;k\mathrm{Hz}$.

**Figure 61:**
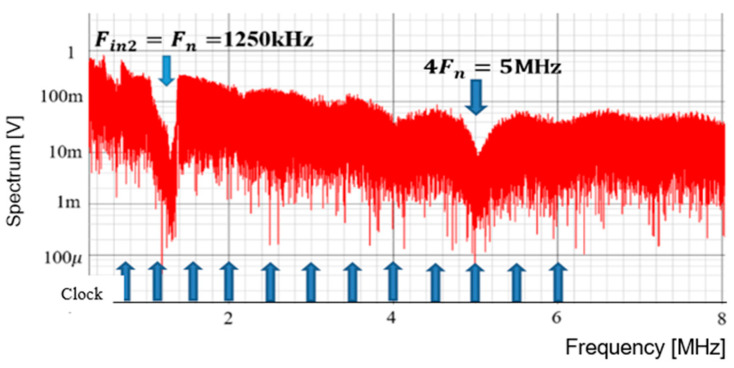
Simulated spectrum for $F_{in2}=1250\;k\mathrm{Hz}$.

Here, we discuss the automatic adjustment of $F_{in}\;$changes from channel 1 to channel 2 in the radio receiver (Figure 59). We set *D* = 0.5, *P* = 1, and the $F_{in}$ of channel 1 to 750 kHz. Then, the output of the automatic PWC controller produces a notch at 750 kHz. In the case that$\;\;F_{in}$ changes to 1250 kHz, the corresponding $F_{ck}$, $W_{H}$, and $W_{L}$ also change, and the notch is automatically produced at 1250 kHz. Simulated spectra are shown in Figure 60 and Figure 61, as $F_{in}$ changes from $F_{n1}$ = 750 kHz to $F_{n2}$= 1250 kHz. The notches are observed at 750 kHz and 1250 kHz, which correspond to $F_{in}$.

### 6.2. Automatic Notch Generation with PWPC Control

This subsection describes the automatic generation of pulse-H, pulse-L, and pulse-LD for the PWPC control.

(A) Automatic Generation Method of PWPC Control

**Figure 62:**
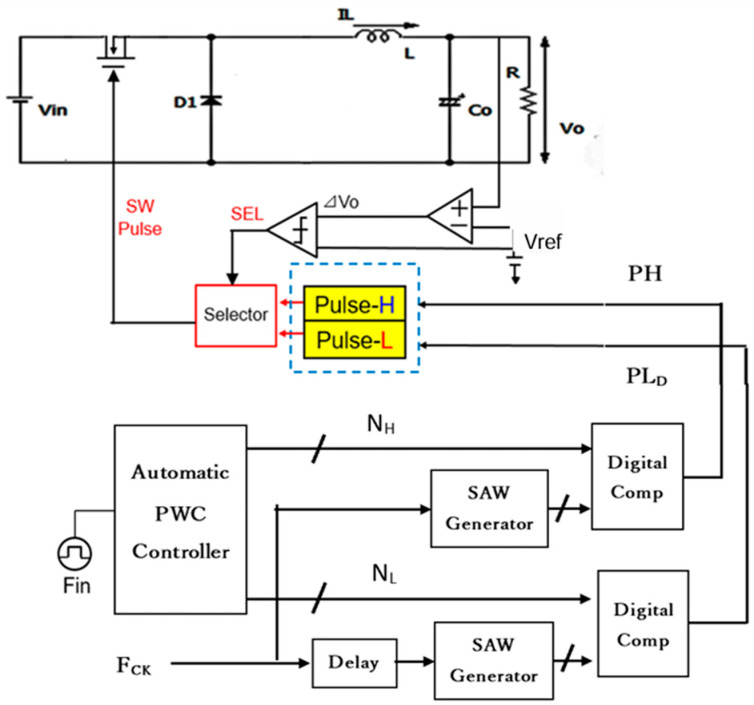
Pulse coding circuit of the PWPC method.

**Figure 63:**
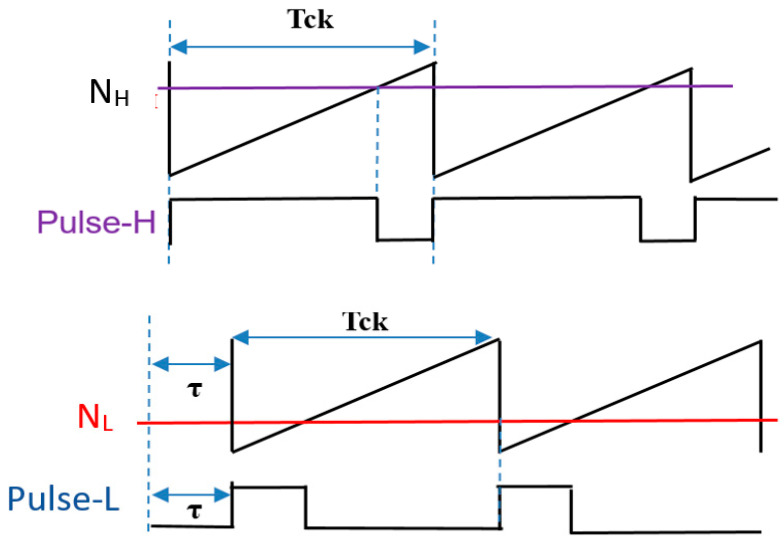
Signals of PWPC control in Figure 62.

In the PWPC method, $F_{n}$ is given by Equations (24) and (27), and Figure 62 illustrates the PWPC configuration. There, the automatic PWC controller generates N_H_ and N_L_. Comparison of N_H_ with the sawtooth waveform produces pulse-H, while comparison of N_L_ with the delayed sawtooth produces pulse-L_D_. Figure 63 shows their waveforms, where the phase shift *τ* is equal to 0.5 $T_{in}$, and Equation (24) is equal to Equation (27) for the steep notch.

The relationship of $F_{ck}$ and $F_{n}$ is given in Equation (44). For *P* = 1, the following is derived from Equation (47). Here, $P_{LD}$ is the timing of the rear end of $P_{L}$.

(51) $$W_{H}=T_{O}+T_{P}=D\;T_{ck}+0.5\;T_{in}\;W_{L}=T_{O}-T_{P}=D\;T_{ck}-0.5\;T_{in}\;P_{LD}=\tau +T_{O}-T_{P}=\tau +D\;T_{ck}-0.5\;T_{in}$$

(B) Simulation Results of Automatic Notch Generation with PWPC Control

**Figure 64:**
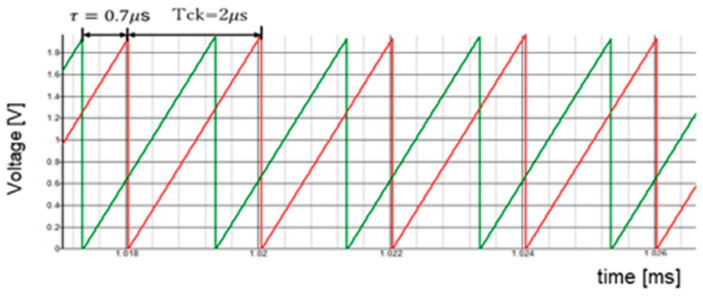
Sawtooth signals with period $T_{ck}$ and delay τ. Green: Original sawtooth. Red: Delayed one.

**Figure 65:**
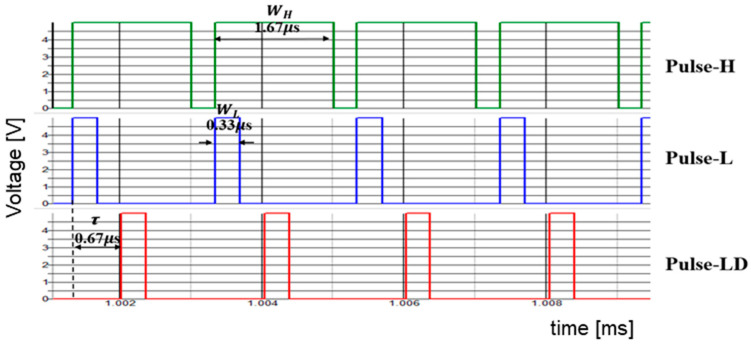
Signals in PWPC circuit.

Figure 64 presents the sawtooth signals, and the primary signals are highlighted in Figure 65. The coding pulses $P_{H}$, $P_{L}$, and $P_{LD}$ are generated by comparing $V_{H}$ and $V_{L}$ with the sawtooth and the delayed sawtooth signals.

There, $V_{in}$ is 10 V, and $\;V_{o}$ is 5 V. When $F_{in}$ is set to 750 kHz and P = 1, $F_{ck}$ is set to 500 kHz. To set $F_{n}$ to $F_{in}$ = 750 kHz, $W_{H}$=1.67 μs,$\;W_{L}=0.33\;\mu s$, and τ = 0.67 μs, based on Equation (51).

**Figure 66:**
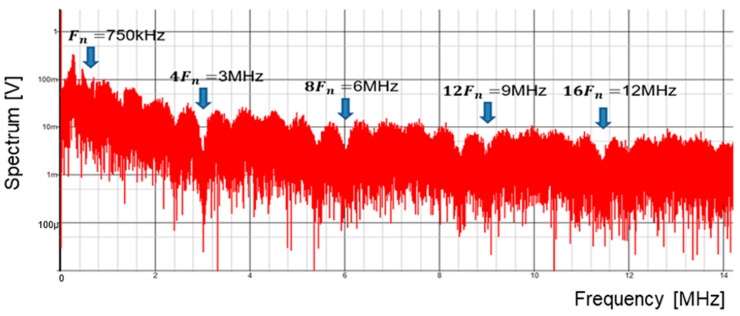
Simulated spectrum with spread spectrum using PWPC method.

Simulation shows that with $W_{H}$ = 1.67 μs, $W_{L}$ = 0.33 μs, and τ = 0.67$\;\mu s,\;\;F_{n}$ is 750 kHz (=$F_{in}$) (Figure 65 and Figure 66). A significant notch appears at 3.0 MHz (=4$F_{n})$. Also, two notches are produced at higher frequencies.

### 6.3. Duty Ratio Generation in Automatic Notch Generation

This subsection discusses a method to automatically detect and set D for $V_{i}$ and $V_{o}$ change.

(A) Analysis of Relationship Between Voltage Conversion Ratio and PWM Duty Ratio

In the automatic PWC control, $F_{in}$ alone can generate $F_{ck}$ as well as $W_{H}$ and $W_{L}$ using Equations (52) and (53) based on Equations (45), (47), and (48). Also see Figure 51. When $D_{H}=D_{L}=D_{P}$ ($D_{P}:\;$shift value of *D*), T_in_ is set to (2/3)T_ck_ for *P* = 1 based on Equation (45).

(52) $$W_{H}=\left(D+D_{H}\right)\;T_{ck}=D\;T_{ck}+0.5\;T_{in}=(D+\frac{1}{3})\;\;T_{ck}$$

(53) $$W_{L}=\left(D-D_{L}\right)\;T_{ck}=D\;T_{ck}-0.5\;T_{in}=(D-\frac{1}{3})\;\;T_{ck}$$

When D varies, the duty cycle of SEL, $D_{s}$ is affected, which influences $\Delta V_{o}$, and the resulting duty ratio $D^{\prime }$ is represented by Equation (54). During the circuit design, the *V_o_/V_i_* ratio is fixed, meaning D, $D_{H}$, and $D_{L}$ are set. Even if $F_{in}\;$is changed, $D_{H}$ and $D_{L}$ are still produced automatically by the circuit. However, when *V_o_* is changed, D also is changed, which differs from the designed D.

For instance, for $T_{in}=0.67\;\mu s$ and $T_{ck}=1\;\mu s$, we set *D = V_o_*/*V_i_ =* 5 *V*/10 *V* = 0.5. That is, for $W_{H}=0.83$ and $W_{L}=0.17$ based on Equations (52) and (53), and $D_{s}$ = 0.5, the waveform of $W_{H}$ and $W_{L}$ remains balanced. However, when D varies, $W_{H}$ changes to 0.86, and $W_{L}$ changes to 0.20. In the designed circuit, when $D_{s}$ remains at 0.5, $W_{H}$ is increased while $W_{L}$ is decreased.

In Equation (54), ∆*D* represents the variation of *D*, and the change rate is defined as x = Δ*D*/*D*. Then, the changed $W_{H}^{\prime }$ and $D_{H}^{\prime }$, along with $W_{L}^{\prime }$ and $D_{L}^{\prime }$, are given by Equations (55) to (58).

(54) $$D^{\prime }=D+\Delta D=D+D\;\frac{\Delta D}{D}=D\;\left(1+x\right)$$

(55) $${W^{\prime }}_{H}=\left(D+\Delta D+D_{H}\right)\;T_{ck}$$

(56) $${D^{\prime }}_{H}=D_{H}-\Delta D\;\to \;D\;\left(1-x\right)$$

(57) $${W^{\prime }}_{L}=\left(D+D-D_{L}\right)\;T_{ck}$$

(58) $${D^{\prime }}_{L}=D_{L}+\Delta D\;\to \;D\;\left(1+x\right)$$

Before *D* varies, $D_{s}$ = 0.5 and $D_{H}:D_{L}=1:1$. This means that the select signal keeps $W_{H}$ and $W_{L}\;$balanced. After *D* varies, $D_{H}^{\prime }:D_{L}^{\prime }$ can be given by Equation (59). The average voltage of the SEL signal, $V_{SEL}$, is given by Equation (60), and $V_{SEL}$ is influenced by $\Delta D_{o}$. Consequently, when $V_{SEL}$ changes, $\Delta V_{o}$ increases.

(59) $${D^{\prime }}_{H}:{D^{\prime }}_{L}=\left(1-x\right):\;\left(1+x\right)$$

(60) $$V_{SEL}=\frac{V_{cc}\left(1-x\right)}{\left(1-x\right)+\left(1+x\right)}=\frac{V_{cc}\left(1-x\right)}{2}=\frac{V_{cc}\left(1-\frac{\Delta D}{D}\right)}{2}$$

It is inferred that when the duty ratio shifts from *D* to *D’*, $D_{H}$ changes to $D_{H}^{\prime }$ while $D_{L}$ changes to $D_{L}^{\prime }$. Consequently, the select signal for $W_{H}$ and $W_{L}$ is no longer balanced. It influences V_SEL_, shifting it from V_cc_/2 to V_cc_ (1 − Δ*D*/*D*)/2. Consequently, the output voltage also increases.

(B) Simulation Results of Duty Ratio Change

**Figure 67:**
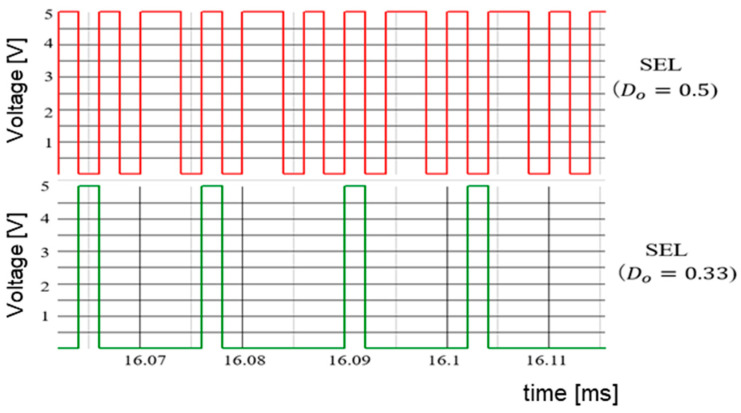
Waveforms of SEL [27] @IEICE.

**Figure 68:**
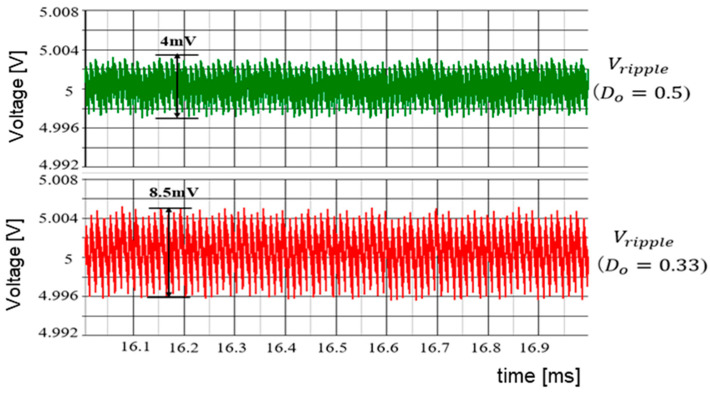
Output voltage ripples [38] @IEEE.

According to Section 5.2, when $V_{i}$ is varied while keeping $W_{H}$ and $W_{L}$ fixed, the duty cycle of SEL changes significantly. This causes significant variations in $I_{L}$ and $\Delta V_{o}$. In simulation, $V_{ref}=V_{o}=$5.0 V and $V_{i}$ are altered to 10 V and 15 V. Correspondingly, *D* changes to 0.5 and 0.33. Figure 67 displays the select signal waveforms. It is observed that for D = 0.5, the waveforms of $W_{H}$ and $W_{L}$ remain balanced. For *D* = 0.33, the waveform of the select signal becomes unbalanced, and the output of $W_{L}$ exceeds that of $W_{H}.$ Figure 68 illustrates $\Delta V_{o}$ as D varies. Any change in *D* affects $\Delta V_{o}$.

(C) Automatic Detection of PWM Duty

Based on *D* and Equations (52) and (53), $W_{H}$ and $W_{L}$ are generated (Figure 50). When $V_{i}$ varies, *D* also varies according to the ratio *D* = V_o_/V_i_. The number of $W_{H}$ and $W_{L}$ pulses are automatically adjusted based on *D*. The detection method of the SAW peak voltage produced by $T_{ck}$ and V_i_ is considered. Under this condition, the peak voltage is proportional to *D*.

**Figure 69:**
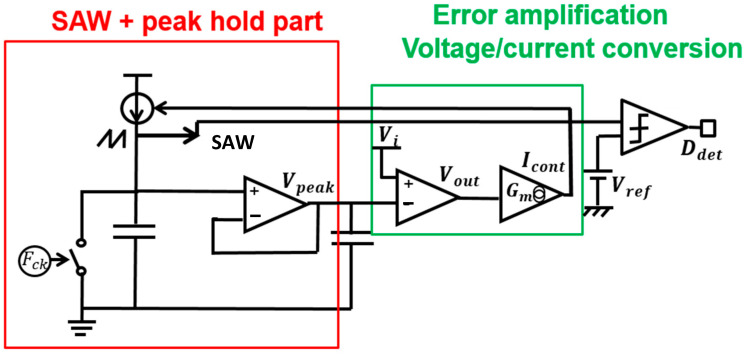
Automatic *D* detection circuit [38] @IEEE.

Figure 69 illustrates the automatic D detection circuit. There, the SAW signal is generated by a current source, with the frequency of the SAW designated as $F_{ck}$. A voltage follower serves as the peak hold circuit, and the peak voltage $V_{peak}$ is compared with $V_{i}\;$by an error amplifier, and an error voltage is generated. Usage of a voltage-controlled current source converts the error voltage into an error current, which is then fed back to the SAW generator. In this process, the SAW peak voltage is automatically detected, which is equal to $V_{i}$. Then, the comparator produces the D detection signal by comparing the SAW signal with $V_{ref}$, which is equal to $V_{o}$.

**Figure 70:**
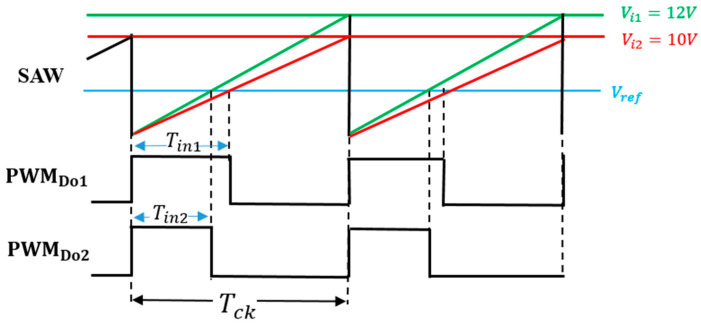
Signals of D detection circuit [38] @IEEE.

Figure 70 shows signal waveforms of the D detection circuit. For $V_{i}=12V$, the SAW peak voltage is 12 V. By comparing the SAW with $V_{ref}$, the sampled data correspond to $D_{1}$. For $V_{i}\;$= 10 V, the SAW peak voltage automatically changes to 10 V. By comparing the SAW and $V_{ref}$, the sampled data become $D_{2}$.

**Figure 71:**
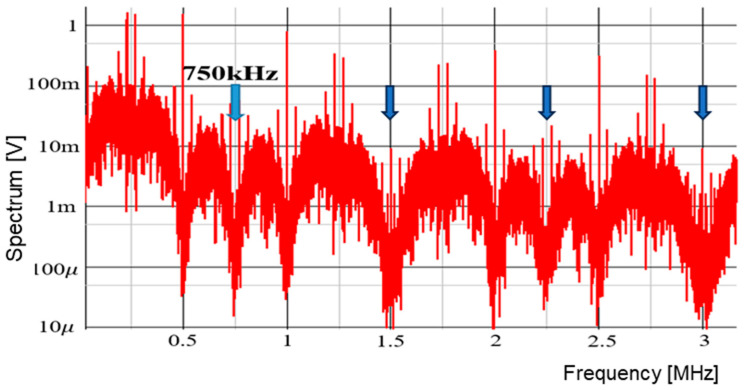
Simulated spectrum with automatic generation without spread spectrum.

We have developed an automatic notch frequency generator using this method. There, D is automatically detected in response to a $V_{i}$ change for the notch creation at the input frequency. The simulation results are shown in Figure 71, with the same parameters as those in Section 5.1, except for $V_{i}$, which is 15 V this time. Additionally, the clock is not modulated, and *D* can be automatically detected as 0.33. The simulation results show that the notch is at 750 kHz (=$F_{in}$).

**Figure 72:**
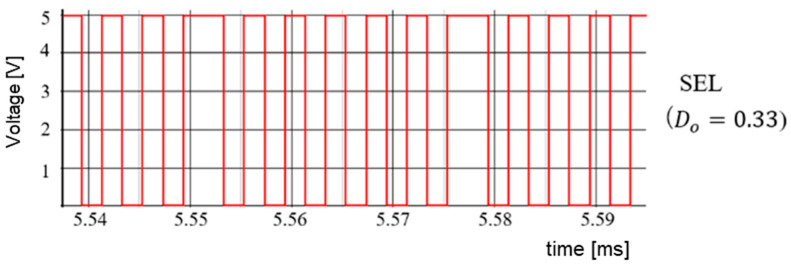
SEL for automatic notch generation.

**Figure 73:**
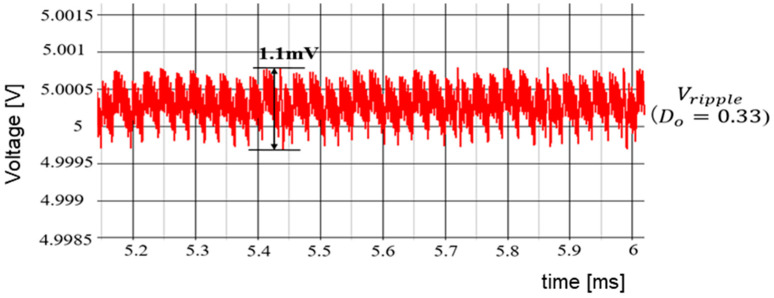
Output voltage ripple in automatic notch generation.

The SEL signal is illustrated in Figure 72. In comparison to Figure 67, under the condition of D = 0.33, the waveform of $W_{H}$ and $W_{L}$ remains balanced. The output voltage ripple is displayed in Figure 73, which is decreased from 8.5 mV to 1.1 mV, compared with Figure 68.

The above discussion says that for setting *D* (0.33 < *D* < 0.67) in automatic notch generation with the PWC control and the D automatic detection method, *D* is detected based on the change in $V_{i}$. This results in a notch being produced at $F_{in}$, while reducing $\Delta V_{o}$.

## 7. Implementation of PWC Controlled Converter with Notch Generation

### 7.1. Experiment of Converter with Notch Generation

**Figure 74:**
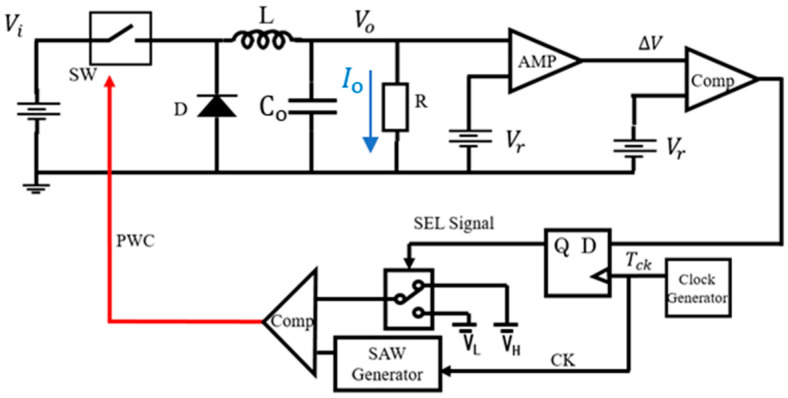
Buck converter with PWC control.

**Figure 75:**
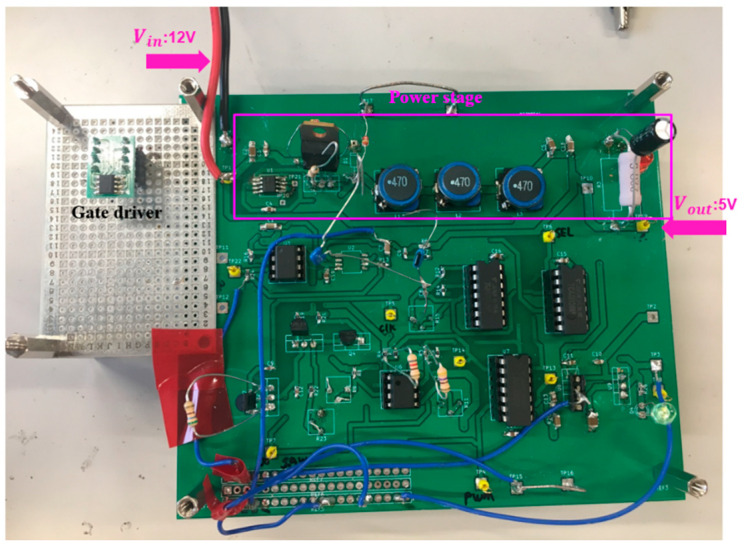
Prototype of PWC control buck converter.

We have verified the notch frequency using the PWC control with the prototype circuit in Figure 74, with the parameters in Table 4. Figure 75 shows its implemented prototype on a PCB board.

**Figure 76:**
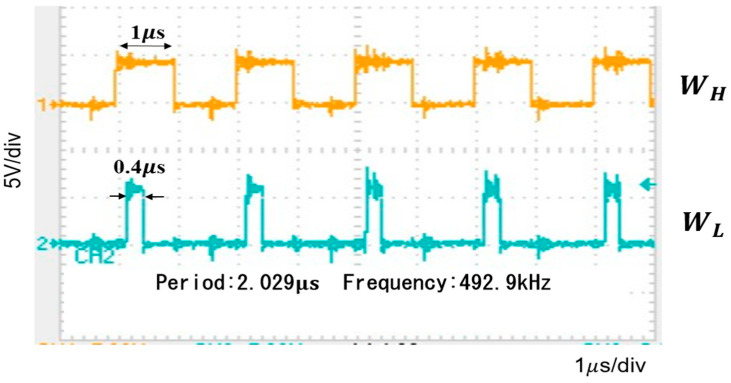
Measured waveforms of $W_{H}$ and $W_{L}$ in the PWC control converter.

**Figure 77:**
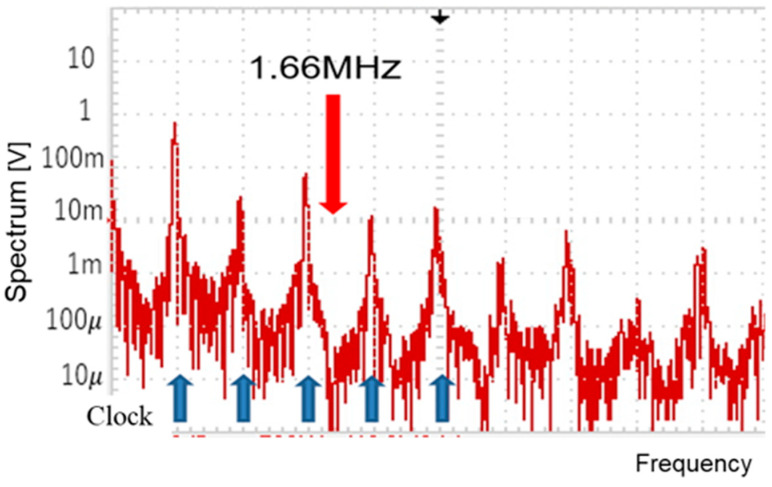
Measured spectrum of the PWC control converter.

Figure 76 shows measured waveforms of $W_{H}$ and $W_{L}$. Additionally, we have analyzed the spectrum of the PWC control converter with $W_{H}=1.0\;\mu s$ and $W_{L}=0.4\;\mu s$ (Figure 77). The notches appear between $F_{ck}$ and 2 $F_{ck}$, between 2 $F_{ck}$ and 3 $F_{ck}$, and between 3 $F_{ck}$ and 4 $F_{ck}$. By substituting the parameter values into Equation (24), 1.66 MHz is obtained, which agrees with the measured result.

### 7.2. Experiment of Automatic Notch Generation

Experiments examine the notch characteristics using the prototype circuit.

(A) Experiment Method of Automatic Notch Generation

**Figure 78:**
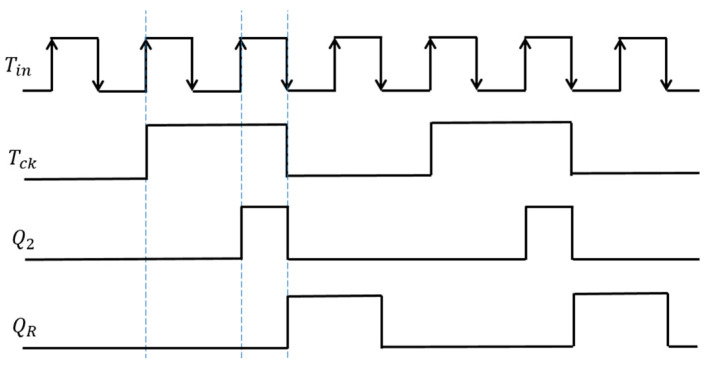
Signals when $T_{in}$ produces $T_{ck}$.

In the automatic notch generation method, the control stage circuit corresponds to the one in Figure 50. We expect that by inputting $F_{in}$, the notch is automatically produced at $F_{in}$. We have $T_{ck}=1.5T_{in}$. In the 1.5$\;F_{in}$ frequency generation circuit (pink border), inputting a pulse with a period of $T_{in}$ produces $T_{ck}$. The waveforms of $T_{in}$, $T_{ck}$, $Q_{2}$, and $Q_{R}$ are illustrated in Figure 78. By comparing the sawtooth waveform with $T_{ck}$ to $V_{H}$ and $V_{L}$, $W_{H}$ and $W_{L}$ are generated, respectively.

**Figure 79:**
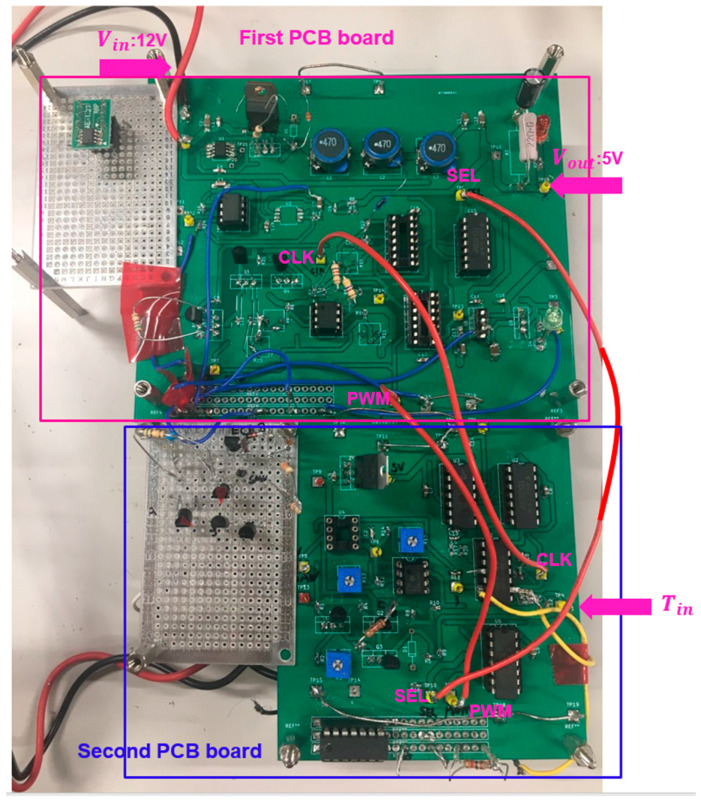
Prototype for automatic notch generation.

Figure 79 shows the prototype with three red wires connecting the two boards. By inputting a pulse with a period of $T_{in}$ using a pulse generator, a notch is produced automatically at the same frequency. Next, we evaluate the prototype circuit with the parameters in Table 5.

**Figure 80:**
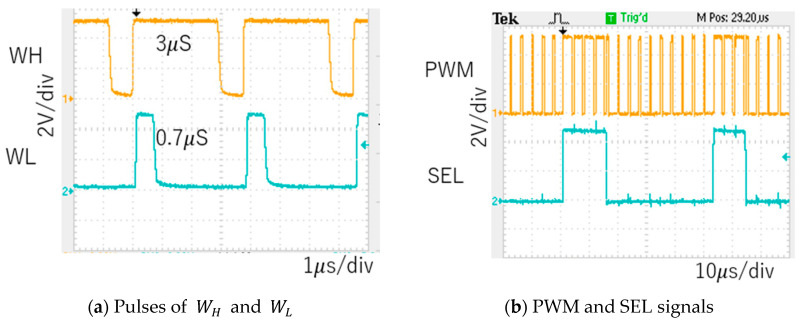
Measured waveforms ($F_{in}=400\mathrm{kHz}$).

For the first example, we set *P* = 1 in Equation (44), and a notch is produced between $F_{ck}$ and 2 $F_{ck}$. We input $F_{in}$= 400 kHz, and $F_{ck}$ can be automatically about 267 kHz ($T_{ck}=3.7\;\mu s$), and $W_{L}=0.7\;\mu s$ (Figure 80a). The PWM and SEL signals are shown in Figure 80b. Based on Equation (24), the notch frequency is calculated as 435 kHz. From the experimental spectrum, the observed notch frequency is approximately 400 kHz, which closely matches the theoretical result by Equation (24) and lies between $F_{ck}$ and 2 $F_{ck}$.

(B) Experimental Results of Automatic Notch Generation

**Figure 81:**
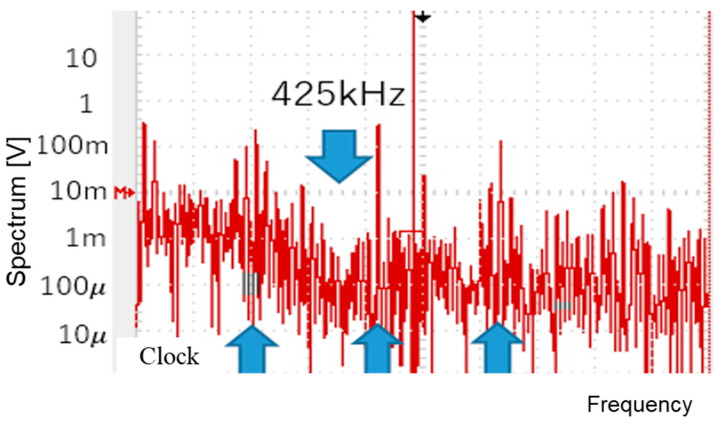
Measured spectrum of PWM signal ($F_{in}=600\mathrm{kHz}$).

When $F_{in}\;$is 400 kHz, $F_{ck}$ is automatically set to 267 kHz ($T_{ck}$ = 3.7 μs) for P = 1. The pulse widths are automatically adjusted to $W_{H}$ = 3.0 μs and $W_{L}$ = 0.7 μs (Figure 80a). The PWM and SEL signals are illustrated in Figure 80b. $F_{n}$ can be calculated as 435 kHz based on Equation (30). The experiments show that the observed $F_{n}$ is around 425 kHz (Figure 81), which matches the theoretical result in Equation (30), and that this notch lies between $F_{ck}$ and $2\;F_{ck}$.

**Figure 82:**
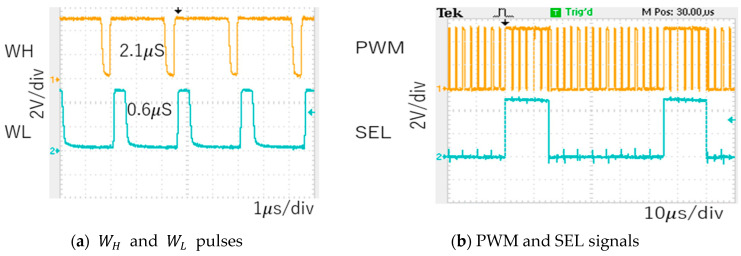
Measured waveforms ($F_{in}=600\mathrm{kHz}$).

**Figure 83:**
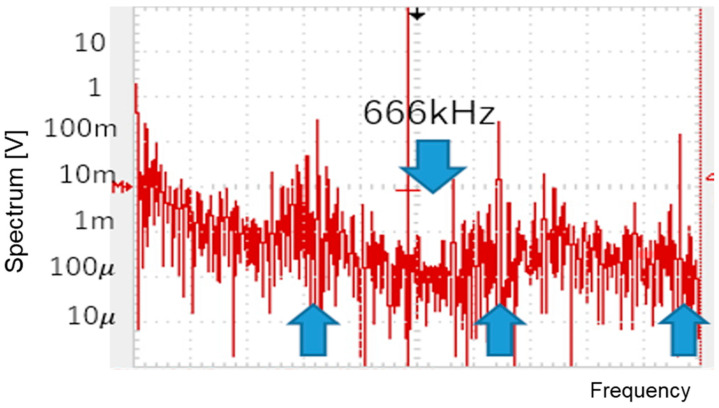
Measured spectrum of PWM signal ($F_{in}=600\;\mathrm{kHz}$).

For the second example, P = 1 is used. By changing $F_{in}$ to 600 kHz, $F_{ck}$ can be automatically adjusted to 400 kHz ($T_{ck}=2.5\;\mu s$). Consequently, $W_{H}=2.1\;\mu s$ and $W_{L}=0.6\;\mu s$ (Figure 82a). The PWM and SEL signals are illustrated in Figure 82b. $F_{n}$ is calculated as 666 kHz based on Equation (24), and the experimental results show that the observed notch is at 666 kHz (Figure 83), which lies between $F_{ck}$ and $2F_{ck}$.

## 8. Discussion on Applications to Sensor Systems

This section discusses possible applications of the reviewed band-selective noise spread spectrum technologies to sensor systems. This paper addresses the noise issues in DC–DC converters, which are a critical concern for every sensor system designer. In particular, it focuses on the signal band noise problems affecting the receiver [46,47,48]. The receiver can be considered a type of sensor system.

The reviewed technology is a switching power supply with notch characteristics, which does not generate noise (spectrum) near the reception frequency for weak radio wave receivers. Specifically, it minimizes noise spectra at specific frequencies in conducted and radiated noise.

In high-performance, compact, lightweight, and low-cost transmission and reception devices, such as cellular transceivers [46,47,48] or wildlife tracking equipment [49,50,51], it is possible to eliminate the need for power noise prevention components like power noise covers or filter circuits. This contributes significantly to the miniaturization, lightweight design, and cost efficiency of these devices.

Providing a switching power supply for receiver systems is frequently used in amateur radio or aviation-related wireless communications [52], where the switching of reception frequencies (channel switching) occurs frequently. This power supply automatically adjusts the notch frequency of the noise spectrum in response to changes in the reception’s primary frequency

Portable sensor systems capable of receiving and detecting weak radio waves hold great potential for applications of the reviewed technology. Here are some representative examples [46,47,48,53,54,55,56,57,58]:

1. **Wireless communication monitoring**: small sensors are used to detect weak signals from technologies such as Wi-Fi, Bluetooth, and NFC. This allows for monitoring the health of communication environments and identifying abnormalities;
2. **Radio wave leakage detection:** this system can be utilized in highly confidential environments to detect radio waves leaking externally and prevent information breaches;
3. **Frequency identification:** detecting weak radio signals and pinpointing their sources or frequency bands can assist in investigations aimed at reducing radio interference;
4. **Smart home appliance management:** an application that detects weak radio waves emitted by smart devices within the household using sensors, allowing for the management of device operational status and connection status;
5. **Security and surveillance system:** detect suspicious signals to monitor unauthorized use of drones or communication devices, enhancing overall security;
6. **Healthcare:** detecting environmental electromagnetic waves (e.g., EMF: Electromagnetic Field) that may affect the human body to aid in environmental management and health protection;
7. **Scientific investigation:** detecting extremely weak signals in the environment has the potential to lead to new discoveries in fields such as space exploration, geology, and meteorology;
8. **IoT (Internet of Things):** sensors detect the faint signals generated by IoT devices, enabling the optimization of networks and efficient energy management.

The advantages of portable sensor systems lie in their ease of transportation and flexible installation. Since each application requires specific technologies and requirements, it is essential to design them tailored to the target use.

## 9. Conclusions

This paper reviews technologies to realize the selectable band in the EMI noise spread spectrum of the DC–DC switching converter; their fundamental circuit, principles, theoretical analysis, simulation, and measurement results are presented. The EMI noise is diffused from the switching signals caused by the clock, and then, the noise spread spectrum techniques with the selectable notch band at the undesirable frequency such as the receiving radio signal band have been developed, while conventional methods may allow noise to encroach upon the signal band.

We review the Pulse Coding Driving (PCD) control method in the selectable notch frequency noise spread spectrum technology. It controls the output voltage using two different pulse counts such as the pulse width coding (PWC) method, pulse phase coding (PPC) method, and a combination of pulse width and phase coding methods. The PCD control can be used in conjunction with the PWM control.

Also, the theoretical derivation of their notch frequencies is shown. The derived equations represent the Fourier transform of the pulse-coded signals, rather than those of the entire converter system. However, the notch frequencies obtained from both the simulation and experimental results align closely with the derived theoretical equations. The derivation of equations for the complete converter will be addressed in future work. Furthermore, the comparison of noise spectrum shapes between simulation and measurement results should be performed with precision, although it may be influenced by various practical circuit parameters, such as the clock slew rate and parasitic effects. Additionally, as future work, a quantitative comparison with conventional methods concerning the noise spread spectrum effect should be conducted.

Further, a PCD control method that automatically tracks changes in the receiving frequency and the input voltage is introduced. The receiving frequency is often switched in the radio receiver, and the input frequency fluctuates. There, the notch band characteristics need to automatically switch to another reception band. We explain a method to detect the received frequency and automatically switch the clock frequency to the proper one, as well as a method so that, for the input voltage change, the PCD control automatically sets two types of pulse counts, while the conventional method automatically controls the duty of the PWM signal.

Finally, we review a PWC control method for automatic notch generation, with the notch characteristics of two types of PCD pulses and their spectra. There, the PCD pulses and the switching of notch frequencies are changed when the reception frequency is switched.

The expansion to the FM and higher frequency bands for the notch and the application to various converters such as single-inductor multi-output converters [59,60,61,62] as well as its implementation in sensor systems are anticipated to be future challenges. Also, IC implementation of the reviewed methods is a next work.

## Figures and Tables

**Table 1:** Simulation parameters.

Parameter	Value	Parameter	Value	Parameter	Value
*V*in	10.0 V	*I*o	0.5 A	*C*o	470 μF
*V*o	5.0 V	*L*	5.0 μH	*F*ck	400 kHz

**Table 2:** Parameters of PWC control simulation.

Parameter	Value	Parameter	Value	Parameter	Value
*V*i	12.0 V	*L*	200 μH	*W*H	1.6 μs
*V*o	5.0 V	*C*	470 μF	*W*L	0.3 μs
*I*o	0.52 A	*T*ck	2.0 μs		

**Table 3:** Parameters of the simulation circuit, which includes the buck converter power stage in Figure 1, along with the PCC control circuit in Figure 33 and the waveforms in Figure 34 and Figure 35.

Parameter	Value	Parameter	Value	Parameter	Value
*V*i	10.0 V	*L*	100 μH	*T* *L*	600 ns
*V*o	3.0 V	*C*	470 μF	*T* *S*	220 ns
*I*o	0.5 A	*Wo*	170 ns		

**Table 4:** Parameter values of Figure 72.

**Parameter**	**Value**	**Parameter**	**Value**	**Parameter**	**Value**
*V*i	12.0 V	*I*o	0.2 A	*C*o	47 μF
*V*o	5.0 V	*L*	100 μH	*T*ck	2 μs

**Table 5:** Parameters of prototype.

Parameter	Value	Parameter	Value	Parameter	Value
*V*i	10.0 V	*I*o	0.16 A	*C*	570 μF
*V*o	3.5 V	*L*	141 μH		

## References

[1] Harada K., Ninomiya T., Gu B. (1992). Circuit Scheme of PWM Converter in Basics of Switching Converter.

[2] Stratakos A.J., Sullivan C.R., Sandersand S.R., Broderson R.W. (1999). High-Efficiency Low-Voltage DC-DC Conversion for Portable Applications in Low-Voltage/Low-Power Integrated Circuits and Systems.

[3] Trzynadlowski A.M., Wang Z., Nagashima J.M., Stancu C., Zelechowsk M.H. (2003). Comparative Investigation of PWM Techniques for a New Drive for Electric Vehicles. IEEE Trans. Ind. Appl..

[4] Erickson R.W., Maksimović D. (2020). Fundamentals of Power Electronics.

[5] Taniguchi K., Sato T., Nabeshima T., Nishijima K. Constant Frequency Hysteretic PWM Controlled Buck Converter. Proceedings of the International Conference on Power Electronics and Drive Systems (PEDS).

[6] Lai R., Maillet Y., Wang F., Wang S., Burgos R., Boroyevich D. (2010). An Integrated EMI Choke for Differential-mode and Common-mode Noise Suppression. IEEE Trans. Power Electron..

[7] Stankovic A.M., Verghese G.C., Perreault D.J. (1995). Analysis and Synthesis of Randomized Modulation Schemes for Power Converters. IEEE Trans. Power Electron..

[8] Tse K.K., Chung H.S., Hui S.Y., So H.C. (2000). Analysis and Spectral Characteristics of a Spread-Spectrum Technique for Conducted EMI Suppression. IEEE Trans. Power Electron..

[9] Giral H., Aroudi E.A., Martinez-Salamero L., Leyva R., Maixe J. (2001). Current Control Technique for Improving EMC in Power Converters. Electron. Lett..

[10] Yuan B., Liang C., Li Z., Zhang Q. A Clock Generator with Dual Pseudo Random Spread Spectrum in DC-DC Buck Converter. Proceedings of the IEEE International Conference on Integrated Circuits, Technologies and Applications (ICTA).

[11] Mihali F., Kos D. (2006). Reduced Conductive EMI in Switched-mode DC–DC Power Converters without EMI Filters: PWM versus Randomized PWM. IEEE Trans. Power Electron..

[12] Li H., Tang W.K.S., Li Z., Halang W.A. (2008). A Chaotic Peak Current Mode Boost Converter for EMI Reduction and Ripple Suppression. IEEE Trans. Circuits Syst. II Express Briefs.

[13] Kao Y.-H., Hung C.-S., Chang H.-H., Guo B., Tsai Y. A 48V-to-5V Buck Converter with Triple EMI Suppression Circuit Meeting CISPR 25 Automotive Standards. Proceedings of the IEEE International Solid-State Circuits Conference.

[14] Fishta M., Raviola E., Fiori F. (2024). EMI Reduction at the Source in WBG Inverters: A Comparative Study of Spread-Spectrum Modulation and Auxiliary Switching Leg Techniques. IEEE Trans. Electromagn. Compat..

[15] Stok E., Otten M., Huisman H., Kösesoy Y. EMI Reduction in an Interleaved Buck Converter Through Spread Spectrum Frequency Modulation. Proceedings of the 25th European Conference on Power Electronics and Applications (EPE’23 ECCE Europe).

[16] Sun T.-W., Li M.-Z., Tsai T.-H., Chang C.-C. A High-Accuracy Hysteretic DC-DC Converter Using a Spread-Spectrum EMI Suppression Technique with Double Gold Codes. Proceedings of the 21st IEEE Interregional NEWCAS Conference (NEWCAS).

[17] Barbaro A., Fishta M., Raviola E., Fiori F. A Comparison of Spread Spectrum and Sigma Delta Modulations to Mitigate Conducted EMI in GaN-Based DC-DC Converters. Proceedings of the 14th International Workshop on the Electromagnetic Compatibility of Integrated Circuits (EMC Compo).

[18] Kundrata J., Baric A. (2024). Clock Frequency Optimization of a Compensated Spread-Spectrum Controller in Buck Converters. IEEE Access.

[19] Kapat S. (2016). Reconfigurable Periodic Bi-frequency DPWM with Custom Harmonic Reduction in DC-DC Converters. IEEE Trans. Power Electron..

[20] Kundrata J., Barić A. Implementation of Voltage Regulation in a Spread-Spectrum-Clocked Buck Converter. Proceedings of the 46th MIPRO ICT and Electronics Convention (MIPRO).

[21] Ioinovici A. (2001). Switched-Capacitor Power Electronics Circuits. IEEE Circuits Syst. Mag..

[22] Li P., Bazzi A., Zhang Z. High-Performance Control of Battery-Interfacing Cascade Buck-Boost Converter. Proceedings of the IEEE Transportation Electrification Conference and Expo.

[23] Tanaka T., Ninomiya T., Harada K. Random-Switching Control in DC-DC Converters. Proceedings of the IEEE PESC.

[24] Tanaka T., Ninomiya T. Random-Switching Control for DC-DC Converters: Analysis of Noise Spectrum. Proceedings of the IEEE 23rd Power Electronics Specialists Conference.

[25] Tanaka T., Hamasaki H., Yoshida H. Random-Switching Control in DC-to-DC Converters: An Implementation Using M-Sequence. Proceedings of the Power and Energy Systems in Converging Markets.

[26] Lin Y., Hsu C., Lin Y.-D. (2020). A Low EMI DC-DC Buck Converter with a Triangular Spread-Spectrum Mechanism. Energies.

[27] Iraheta A. (2021). Further Optimizing EMI with Spread Spectrum.

[28] Curtis P., Lee E. (2022). EMI Reduction Technique, Dual Random Spread Spectrum.

[29] Pareschi F., Rovatti R., Setti G. (2015). EMI Reduction via Spread Spectrum in DC/DC Converters: State of the Art, Optimization, and Tradeoffs. IEEE Access.

[30] Leonard J. (2004). Dual Switcher with Spread Spectrum Reduces EMI. Linear Technol. Mag..

[31] Zimmer G., Scott K. Spread Spectrum Frequency Modulation Reduces EMI, Technical Article, Analog Devices. https://www.analog.com/en/resources/technical-articles/spread-spectrum-frequency-modulation-reduces-emi.html.

[32] Jaffe S. (2021). The Pros and Cons of Spread-Spectrum Implementation Methods in Buck Regulators. Analog. Des. J..

[33] Miki N., Tsukiji N., Asaishi K., Kobori Y., Takai N., Kobayashi H. EMI Reduction Technique With Noise Spread Spectrum Using Swept Frequency Modulation for Hysteretic DC-DC Converters. Proceedings of the IEEE International Symposium on Intelligent Signal Processing and Communication Systems (ISPACS).

[34] Oiwa N., Sakurai S., Sun Y., Tri M.T., Li J., Kobori Y., Kobayashi H. EMI Noise Reduction for PFC Converter with Improved Efficiency and High Frequency Clock. Proceedings of the IEEE 14th International Conference on Solid-State and Integrated Circuit Technology.

[35] Kobori Y., Sun Y., Tri M.T., Kuwana A., Kobayashi H. (2020). EMI Reduction in Switching Converters With Pseudo Random Analog Noise. J. Technol. Soc. Sci..

[36] Kobori Y., Arafune T., Tsukiji N., Kobayashi H. Selectable Notch Frequencies of EMI Spread Spectrum Using Pulse Modulation in Switching Converter. Proceedings of the IEEE 11th International Conference on ASIC.

[37] Sun Y., Kobori Y., Kobayashi H. Full Automatic Notch Generation in Noise Spectrum of Pulse Coding Controlled Switching Converter. Proceedings of the IEEE 14th International Conference on Solid-State and Integrated Circuit Technology.

[38] Sun Y., Kobori Y., Kuwana A., Kobayashi H. (2020). Pulse Coding Controlled Switching Converter that Generates Notch Frequency to Suit Noise Spectrum. IEICE Trans. Commun..

[39] Dong G., Katayama S., Sun Y., Kobori Y., Kuwana A., Kobayashi H. Notch Frequency Generation Methods in Noise Spread Spectrum for Pulse Coding Switching DC-DC Converter. Proceedings of the 13th Latin American Symposium on Circuits and Systems.

[40] Gamoudi R., Chariag D.E., Sbita L. (2018). A Review of Spread-Spectrum-Based PWM Techniques—A Novel Fast Digital Implementation. IEEE Trans. Power Electron..

[41] Jankovskis J., Stepins D., Tjukovs S., Pikulins D. (2008). Examination of Different Spread Spectrum Techniques for EMI Suppression in dc/dc Converters. Elektron. Ir Elektrotechnika.

[42] Dousoky G.M., Shoyama M., Ninomiya T. (2011). FPGA-Based Spread-Spectrum Schemes for Conducted-Noise Mitigation in DC–DC Power Converters: Design, Implementation, and Experimental Investigation. IEEE Trans. Ind. Electron..

[43] Hegarty T. (2018). An Overview of Radiated EMI Specifications for Power Supplies.

[44] Hegarty T. (2018). An Overview of Conducted EMI Specifications for Power Supplies.

[45] Hegarty T. (2018). A Review of EMI Standards, Part 2—Radiated Emissions.

[46] Pozar D.M. (2011). Microwave Engineering.

[47] Razavi B. (2011). RF Microelectronics.

[48] Rappaport T.S. (2024). Wireless Communications: Principles and Practice.

[49] Pereira E., Araújo Í., Silva L.F.V., Batista M., Júnior S., Barboza E. (2023). RFID Technology for Animal Tracking: A Survey. IEEE J. Radio Freq. Identif..

[50] Priyadharsini S., Renukasri V., Sneha R., Sowmiya P.K., Swaathi K. (2020). Wildlife Animal Tracking System using GPS and GSM. Int. J. Eng. Res. Technol..

[51] Kim S.-H., Kim D.-H., Park H.-D. Animal Situation Tracking Service Using RFID, GPS, and Sensors. Proceedings of the IEEE Second International Conference on Computer and Network Technology.

[52] Park P., Di Marco P., Nah J., Fischione C. (2021). Wireless Avionics Intracommunications: A Survey of Benefits, Challenges, and Solutions. IEEE Internet Things J..

[53] Sazonov E. (2020). Wearable Sensors: Fundamentals, Implementation and Applications.

[54] Wilson J.S. (2005). Sensor Technology Handbook.

[55] Roggen D. (2015). Mobile Sensors and Context-Aware Computing.

[56] Khanna V.K. (2024). IoT Sensors—An Exploration of Sensors for Internet of Things.

[57] Pradhan B., Mukhopadhyay S., Sensors I. (2024). AI and XAI: Empowering a Smarter World.

[58] Vermesan O., Friess P. (2022). Internet of Things Applications—From Research and Innovation to Market Deployment.

[59] Lee A.T.L., Jin W., Tan S.-C., Hui R.S.Y. (2021). Single-Inductor Multiple-Output Converters: Topologies, Implementation, and Applications.

[60] Rooholahi B., Siwakoti Y.P., Eckel H.-G., Blaabjerg F., Bahman A.S. (2024). Enhanced Single-Inductor Single-Input Dual-Output DC–DC Converter With Voltage Balancing Capability. IEEE Trans. Ind. Electron..

[61] Zhang X., Zhao A., Li X., Jiang Y., Martins R.P., Mak P.-I. An 80W Single-Inductor DC-DC Architecture for Simultaneous Flash Charging and Dual-Output PoL Supply with 92.1% Peak Efficiency from 15V-to-28V Input to 12.6V/3.3V/1V Outputs Using 1.3 mm3 Inductor. Proceedings of the IEEE European Solid-State Electronics Research Conference (ESSERC).

[62] Yeh W.-T., Cai M.-X., Tsai C.-W., Tsai C.-H. (2025). A Single-Inductor Bipolar-Output DC-DC Converter With Tunable Asymmetric Power Distribution Control (APDC) for AMOLED Applications. IEEE Access.

